# WD repeat domain 5 promotes chemoresistance and Programmed Death-Ligand 1 expression in prostate cancer

**DOI:** 10.7150/thno.55814

**Published:** 2021-03-04

**Authors:** Qianghua Zhou, Xu Chen, Haixia He, Shengmeng Peng, Yangjie Zhang, Jingtong Zhang, Liang Cheng, Sen Liu, Ming Huang, Ruihui Xie, Tianxin Lin, Jian Huang

**Affiliations:** 1 Department of Urology, Sun Yat-sen Memorial Hospital, Sun Yat-sen University, Guangzhou 510120, China.; 2 Guangdong Provincial Key Laboratory of Malignant Tumor Epigenetics and Gene Regulation, Sun Yat-Sen Memorial Hospital, Sun Yat-Sen University, Guangzhou 510120, China.; 3 State Key Laboratory of Oncology in South China & Collaborative Innovation Center of Cancer Medicine, Sun Yat-sen University Cancer Center, Guangzhou, 510060, China.; 4 Department of Medical Oncology, Sun Yat-sen University Cancer Center, Guangzhou, 510060, China.; 5 Department of Urology, The Affiliated Kashi Hospital, Sun Yat-sen University, Kashi, China.

**Keywords:** Prostate cancer, WDR5, chemoresistance, OICR-9429, PD-L1

## Abstract

**Purpose:** Advanced prostate cancer (PCa) has limited treatment regimens and shows low response to chemotherapy and immunotherapy, leading to poor prognosis. Histone modification is a vital mechanism of gene expression and a promising therapy target. In this study, we characterized WD repeat domain 5 (WDR5), a regulator of histone modification, and explored its potential therapeutic value in PCa.

**Experimental Design:** We characterized specific regulators of histone modification, based on TCGA data. The expression and clinical features of WDR5 were analyzed in two dependent cohorts. The functional role of WDR5 was further investigated with siRNA and OICR-9429, a small molecular antagonist of WDR5, *in vitro* and *in vivo*. The mechanism of WDR5 was explored by RNA-sequencing and chromatin immunoprecipitation (ChIP).

**Results:** WDR5 was overexpressed in PCa and associated with advanced clinicopathological features, and predicted poor prognosis. Both inhibition of WDR5 by siRNA and OICR-9429 could reduce proliferation, and increase apoptosis and chemosensitivity to cisplatin *in vitro* and *in vivo*. Interestingly, targeting WDR5 by siRNA and OICR-9429 could block IFN-γ-induced PD-L1 expression in PCa cells. Mechanistically, we clarified that some cell cycle, anti-apoptosis, DNA repair and immune related genes, including AURKA, CCNB1, E2F1, PLK1, BIRC5, XRCC2 and PD-L1, were directly regulated by WDR5 and OICR-9429 in H3K4me3 and c-Myc dependent manner.

**Conclusions:** These data revealed that targeting WDR5 suppressed proliferation, enhanced apoptosis, chemosensitivity to cisplatin and immunotherapy in PCa. Therefore, our findings provide insight into OICR-9429 is a multi-potency and promising therapy drug, which improves the antitumor effect of cisplatin or immunotherapy in PCa.

## Introduction

Prostate cancer (PCa) is the most commonly diagnosed malignancy and the second leading cause of male cancer-related death in the Western world 1. The advanced PCa is mainly treated with Androgen Deprivation Therapy (ADT) and shows a good disease control initially. However, when the disease progresses to castration-resistant prostate cancer (CRPC), there are limited treatments for those patients and lead to dissatisfied outcomes 2, 3. Although the next-generation antiandrogen Enzalutamide (Enz) exhibit promising inhibitory effect by extending patients survival an extra 4.8 months, PCa patients eventually progress to Enz-resistance latter 4. Additionally, Cisplatin (CDDP) based chemotherapy is not the first-line treatment in CRPC due to dose-limiting toxicities and primary or acquired resistance to CDDP 5, 6. Although immune checkpoint inhibitors (ICIs), in particular blockade of PD-1/PD-L1, show promising therapy response in some cancers, they are less effective in PCa which may be poor infiltration of cytotoxic T-cells 7-9. Therefore, it is an urgent need to develop new and combined therapeutic strategies for advanced PCa.

Epigenetic regulation, including modifications of DNA and histones, is a vital mechanism of gene expression and promising therapy target 10-12. WDR5 is a prominent component of SET1/MLL histone-methyltransferase complex, which mainly catalyzing histone H3 lysine 4 (H3K4) di- and tri-methylation (H3K4me3) 13. H3K4me3 is widely distributed with respect to transcription start sites (TSS) and exerts an activated role of target gene transcriptions 14. In addition, WDR5 is also a critical co-activator for c-Myc recruitment to chromatin and activate transcription, leading to tumorigenesis 15. Aberrant expression of WDR5 was demonstrated in some cancers such as bladder cancer, leukemias and prostate cancer. Furthermore, WDR5 participates in multiple biologic functions, such as proliferation, metastasis, chemo-resistance and AR-mediated castration resistance 16-18. However, the clinical role, biologic function and mechanism of WDR5 in PCa remain largely unknown, especially in combined with chemotherapy and immunotherapy.

OICR-9429 is a highly selective and potent antagonist of interaction of WDR5 with partners, including MLL1 and c-Myc 19. The pharmacological mechanism of OICR-9429 is blocking the interaction between the WIN (WDR5 interaction) site, a peptide-binding pocket, with an arginine-containing motif (WIN motif), which consequently inhibits the catalytic activity of MLL1 and potently suppresses H3K4 methylation. Furthermore, OICR-9429 could also role as WDR5-binding motif (WBM) site inhibitor for blocking the WDR5-c-Myc interaction and consequently thwarting c-Myc function in cancer cells 20. OICR-9429 has displayed antitumor effect in several cancers, such as leukemia, colon cancer and pancreatic cancer 19-21. However, it is unclear whether OICR-9429 could be a novel and effective target therapy drug in PCa.

In present study, we first reveal the clinicopathological relevance and roles of WDR5 in PCa. Next, we confirm the pharmacological effect of OICR-9429 on enhancing chemosensitivity and reversing immunosuppressive microenvironment in PCa. Finally, we demonstrate the mechanisms of WDR5-mediated oncogenic roles via transcriptional activation of target genes by H3K4me3 and c-Myc. The critical aim of this study is to facilitate the translation from *in vitro* and vivo discovery into pre-clinical and clinical trials for the treatment of advanced PCa.

## Results

### High expression of WDR5 is associated with advanced clinicopathological features and poor prognosis in prostate cancer

To identify important epigenetic regulators in PCa, we selected 25 epigenetic regulators of histone methylated modifications, including H3K4, H3K9, H3K27 and H3K36, and analyzed their expression between cancer and adjacent normal tissues in TCGA data. 9 out of 25 epigenetic regulators were overexpressed in prostate tumor tissue compared with matched adjacent normal tissue (Figure S1A-B). Kaplan-Meier analysis further demonstrated that only EZH2 and WDR5 displayed positive correlation with poor prognosis (Figure S1C-D). Previous studies had fully demonstrated the oncogenic roles of EZH2 in PCa. Thus, we determined to investigate the clinical significance and the tumor-promoting mechanisms of WDR5 in PCa in this study.

**Figure 1:**
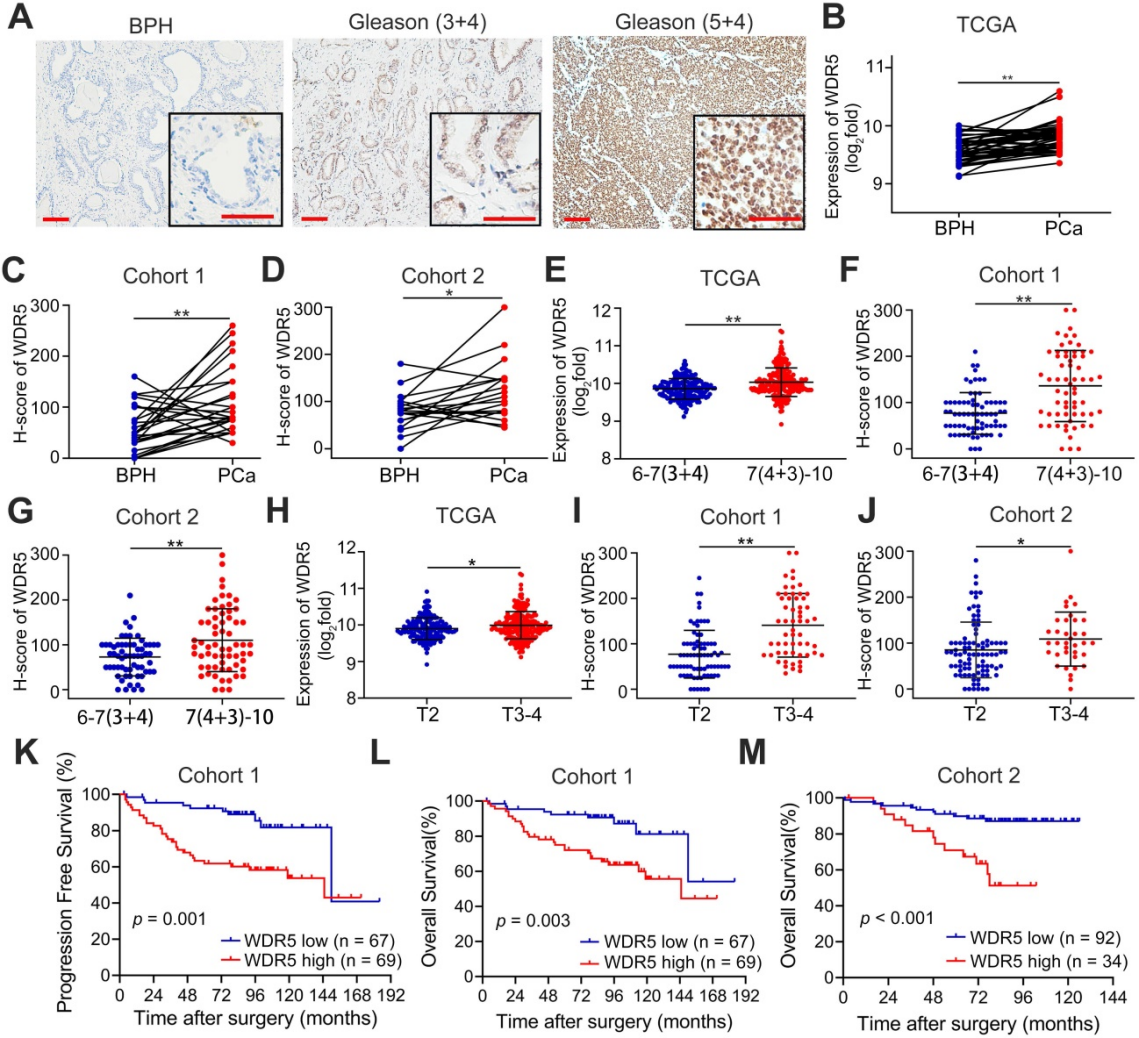
** WDR5 expression correlates with advanced clinicopathological features and predicts poor prognosis. A**. Representative IHC images of WDR5 in BPH and PCa with Gleason (3+4) or Gleason (5+4). Scale bars: red, 50 µm. **B-D**. The expression of WDR5 between BPH and PCa tissues in TCGA cohort (B), Cohort 1(C) and Cohort 2 (D). **E-G**. The expression of WDR5 between PCa tissues with low Gleason (6-7(3+4)) or high Gleason (7(4+3)-10) in TCGA cohort (E), Cohort 1(F) and Cohort 2 (G). **H-J**. The expression of WDR5 between PCa tissues with low T stage (T2) or high T stage (T3-T4) in TCGA cohort (H), Cohort 1(I) and Cohort 2 (J). **K**. Kaplan-Meier curves for Progression free survival of PCa patients with high or low expression of WDR5 in Cohort 1. **L, M**. Kaplan-Meier curves for Overall survival of PCa patients with high or low expression of WDR5 in Cohort 1 (L) and Cohort 2 (M). The error bars mean standard deviations of three independent experiments. *p < 0.05, **p < 0.01.

First, to further determine the clinical relevance of WDR5 in prostate cancer, we analyzed the expression of WDR5 in 262 archived human paraffin-embedded prostate cancer tissues from two cohorts (cohort 1 and cohort 2) using immunochemistry. As shown in Figure 1A-D, a higher level of WDR5 protein was detected in cancer tissues than in adjacent normal or benign prostate tissues. Moreover, overexpression of WDR5 displayed significant association with worse clinicopathological characteristics including advanced Gleason score and T stage in three cohorts (Figure 1E-J). In addition, overexpression of WDR5 was found in patients with lymph node metastases from cohort 1 and TCGA cohort (Figure S2A-B). Kaplan-Meier analysis revealed that overexpression of WDR5 was significantly associated with shorter progression free survival (PFS) in cohort 1 and TCGA (Figure 1K and Figure S1D), and overall survival (OS) in cohort 1 and cohort 2 (Figure 1L-M). A univariate Cox regression analysis demonstrated that patients with high expression of WDR5 had significantly increased risks of disease progression and/or all cause of death in three cohorts (Table S1-2). Furthermore, multivariate analysis showed that overexpression of WDR5 was an independent prognostic factor for PFS in cohort 1 and TCGA (Table S1) and for OS in cohort 1 (Table S2). Taken together, these data demonstrated that WDR5 overexpression was correlated with unfavorable clinicopathological features and prognosis, suggesting that WDR5 might serve as a clinical biomarker for PCa.

### Knockdown WDR5 inhibited proliferation, and enhanced apoptosis and sensitivity to Cisplatin in prostate cancer *in vitro*

**Figure 2:**
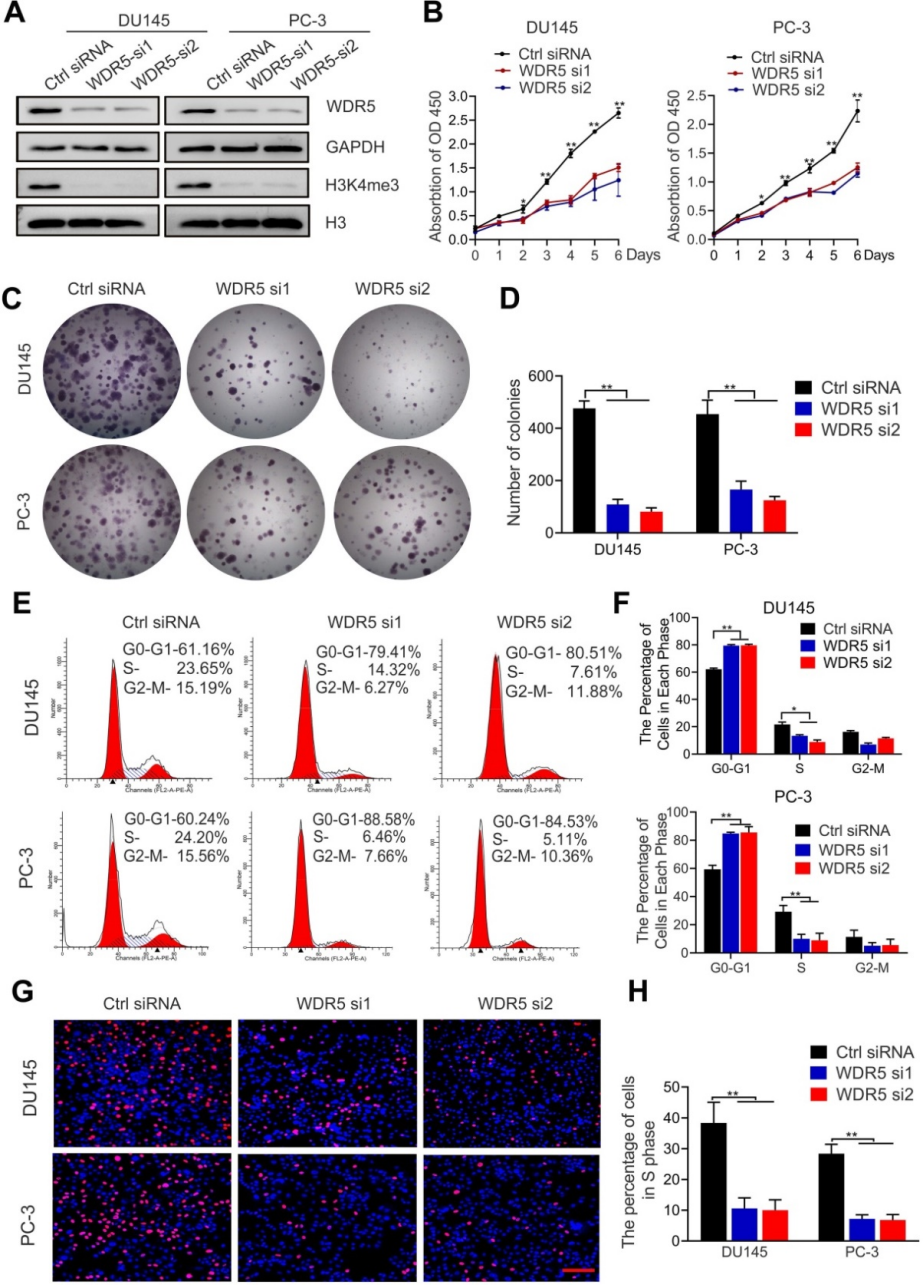
** Knockdown WDR5 inhibits proliferation of PCa cells *in vitro*. A**. Western blot analysis of WDR5 and H3K4me3 expression in WDR5-silenced cells and control cells. GAPDH and H3 were used as internal controls. **B**. MTT assays of DU145 (left) and PC-3 (right) cells transfected with WDR5 or Ctrl siRNA. **C, D**. The images (C) and quantification (D) of colony formation assay of DU145 and PC-3 cells transfected with WDR5 or Ctrl siRNA. **E, F**. Representative images (E) and quantification (F) of cell cycle in DU145 and PC-3 cells transfected with WDR5 or Ctrl siRNA, analyzed by flow cytometry analysis. **G, H**. The images (G) and quantification (H) of EdU assay of DU145 and PC-3 transfected with WDR5 or Ctrl siRNA. Statistical significance was evaluated by using a two-tailed t test or one-way ANOVA. Error bars mean the standard deviations of three independent experiments. *p < 0.05, **p < 0.01.

To explore the function of WDR5 in PCa, we established WDR5-knockdown PC-3 and DU145 PCa cells, and western blotting was performed for confirming the efficiency (Figure 2A). Both MTT and colony formation assays showed that the viability and proliferation of PCa cells were significantly decreased after WDR5 silencing (Figure 2B-D). Furthermore, flow cytometry and EdU assays demonstrated that WDR5-knockdown increased dramatically the percentage of cells in G0/G1 phase and reduced the percentage of cells in S phase in PCa cells (Figure 2E-H). These data indicated that WDR5 enhanced proliferation via regulating G1/S phase transition.

**Figure 3:**
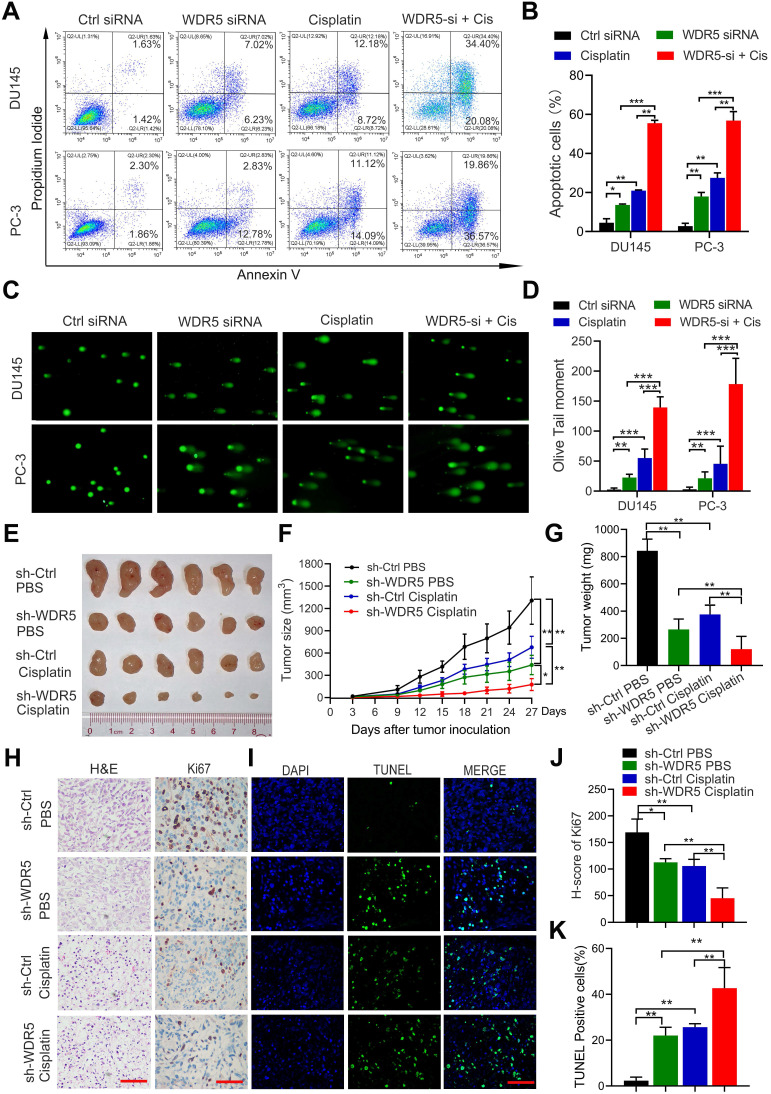
** Inhibition of WDR5 increases chemosensitivity to cisplatin in PCa cells* in vitro* and *in vivo*. A, B**. The images (A) and quantification (B) of cell apoptosis in PCa cells, which were transfected with control or WDR5 siRNA and treated with 1 µg/mL cisplatin for 48 h. **C, D**. Representative images (C) and quantification (D) of comet assay in PCa cells, which were transfected with control or WDR5 siRNA and treated with 1 µg/mL cisplatin for 48 h. **E**. The images of surgically dissected tumors in indicated groups. **F**. The volume of tumors in indicated groups, measured every 3 days. The results are showed as the means ± standard deviation (SD) of values (n = 6). **G**. The weight of tumors in indicated groups were measured after the tumors were surgically dissected. The results are showed as the means ± SD of values (n = 6). **H**. Representative images of Ki67 expression in tumors of indicated groups, examined by IHC. **I**. Representative images of apoptosis in tumors of indicated groups, detected by TUNEL assay. The scale bars in IHC and TUNEL images represent 50 µm. **J, K**. the quantification of the score of Ki67 (J) and percentage of TUNEL-positive cells (K) in indicated groups. *p < 0.05, **p < 0.01.

To further determine the effect of WDR5 on apoptosis, the Annexin V/PI apoptotic assay was performed. The data showed that WDR5 knockdown significantly increased the apoptotic proportion in PCa cells (Figure S3A-B). Considering the importance of epigenetic modification in the chemo-resistance, we next evaluated whether silencing of WDR5 could enhance chemosensitivity to docetaxel or cisplatin in PCa cells. Interestingly, MTT assays revealed that WDR5 depletion dramatically increased the sensitivity of PCa cells to cisplatin, but not docetaxel (Figure S4A-D). The IC_50_ values of cisplatin significantly decreased in PCa cells with WDR5 knockdown, compared with those in respective control cells (Figure S4E). Moreover, the Annexin V/PI apoptotic assay showed that after treatment of the cisplatin, the apoptosis proportion of PCa cells transfected with WDR5 siRNA was significantly increased, compared with control siRNA (Figure 3A-B). Given cisplatin caused cell apoptosis via inducing DNA damage, we then checked whether WDR5 blocked DNA damage caused by cisplatin. Comet assay indicated that silencing WDR5 markedly increased DNA damage degree caused by cisplatin in PCa cells (Figure 3C-D). Taken together, these data revealed that WDR5 was an important regulator in proliferation and chemo-resistance of PCa cells.

### Knockdown WDR5 inhibited tumorigenesis and increased chemosensitivity to Cisplatin in prostate cancer *in vivo*

To further determine the tumorigenesis-promoting and cisplatin-resisting role of WDR5 *in vivo*, subcutaneous xenograft mouse models were established. Stable WDR5 knockdown PC-3 cells or control cells were injected subcutaneously into male nude mice. One week after inoculation, the nude mice bearing PC-3-sh-WDR5 or PC-3-sh-control xenografts were selected randomly for treatment with PBS or cisplatin as reported previously and tumor were measured every three days. As shown in Figure 3E-G, WDR5 knockdown showed a significantly slower tumor growth and smaller tumor weight than control group, and these phenomena were more prominently in cisplatin treatment compared with PBS. Moreover, the proliferation maker Ki67 expression was decreased, but the proportion of apoptotic cells was increased in tumors derived from WDR5 knockdown, and these phenomena were more significantly under cisplatin treatment, compared with corresponding control groups (Figure 3H-K). Collectively, these data demonstrated that targeting WDR5 inhibited PCa cell growth and increased the cisplatin sensitivity *in vivo*.

### OICR-9429 suppresses proliferation, promotes apoptosis and enhances the chemosensitivity of cisplatin in PCa cells

**Figure 4:**
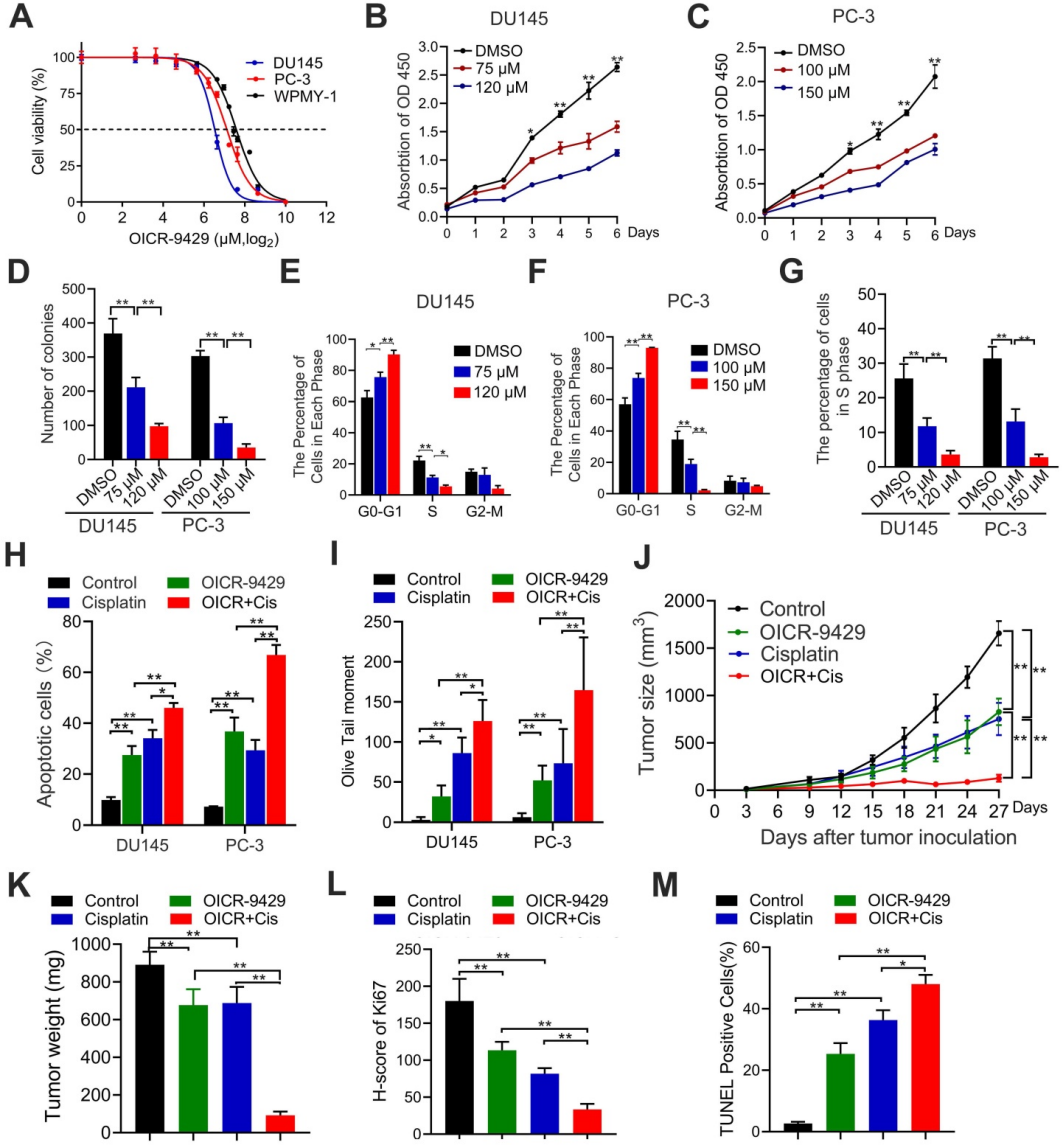
** The WDR5 inhibitor OICR-9429 inhibits proliferation and increases chemosensitivity to cisplatin in PCa cells. A**. Cell viability of DU145, PC-3 and WPMY-1 cells under OICR-9429 treatment. Cells were treated with indicated concentration of OICR-9429 for 48 h and the viability was calculated by MTT assay. Data are showed as mean ± SD of at three independent experiments. **B, C**. MTT assays of DU145 (B) and PC-3 (C) cells treated with OICR-9429 or DMSO at the indicated time points. **D**. Quantification of colony formation assay of DU145 and PC-3 treated with OICR-9429 or DMSO. **E, F**. Quantification of cell cycle in DU145 (E) and PC-3 (F) treated with OICR-9429 or DMSO, analyzed by flow cytometry analysis. **G**. Quantification of EdU assay of DU145 and PC-3 treated with OICR-9429 or DMSO. **H, I**. Quantification of cell apoptosis (H) and Comet assay (I) in PCa cells, which were treated with OICR-9429 (75 µM in DU145 and 100 µM in PC-3) or cisplatin (1 µg/mL) or a combination of both at the same time for 48 h. DU145 cells were on the left, and PC-3 cells were on the right. **J**. The volume of tumors in indicated groups, measured every 3 days. **K**. The weight of tumors in indicated groups measured after the tumors were surgically dissected. The results are presented as the means ± SD of values (n = 6). **L, M**. The quantification of the score of Ki67 (L) and proportion of TUNEL-positive cells (M) in indicated groups. *p < 0.05, **p < 0.01.

Next, we explored the pharmacological effects of OICR-9429, a WDR5 inhibitor, on PCa cells. MTT assays demonstrated that PCa cells viability was significantly decreased by OICR-9429 treatment in a dose-dependent manner (Figure 4A-C). But OICR-9429 was less cytotoxic to normal prostate cells WPMY-1 characterized by low WDR5 expression, and WDR5 knockdown PCa cells, suggesting that OICR-9429 could selectively inhibit PCa cells with a WDR5 dependent manner (Figure 4A and Figure S5A-B). In line with the MTT results, OICR-9429 effectively decreased the colony-formation capacities of PCa cells dose-dependently (Figure 4D and Figure S6A). Moreover, both flow cytometry and EdU assays demonstrated that OICR-9429 induced G_0_/G_1_ phase arrest in PCa cells (Figure 4E-G and Figure S6B-C).

Similarly, OICR-9429 induced a significant dose-dependent apoptosis in PCa cells after 48 h of treatment (Figure S3C-D). To explore whether the combination of OICR-9429 and cisplatin could synergistically inhibit cell viability in PCa cells, the combination index (CI) values were calculated according to the Chou-Talalay median-effect principle 22. Interestingly, OICR-9429 had a synergistic effect with cisplatin on inhibition of PCa cells viability (Figure S7). Moreover, OICR-9429 reinforced the effects of cisplatin on inducing cell apoptosis and DNA damage in PCa cells (Figure 4H-I and Figure S8A-B). Therefore, OICR-9429 inhibits proliferation and enhances the chemo-resistance of PCa cells *in vitro*.

### OICR-9429 inhibits tumorigenesis and enhances cisplatin efficacy in prostate cancer *in vivo*

To further evaluate the pharmacological effects of OICR-9429 *in vivo* model, the wide type PC-3 cells were injected subcutaneously into nude mice. One week after injection, the nude mice were randomly subdivided into four groups and treated with PBS, OICR-9429 alone, cisplatin alone and OICR-9429 combined cisplatin every three days, respectively. As shown in Figure 4J-K and Figure S8C, treatment with OICR-9429 significantly increased the sensitivity of PCa to cisplatin chemotherapy, as indicated by the decreased tumor volume and weight. Notably, compared with tumor treated with cisplatin alone, combined OICR-9429/cisplatin treatment caused a max reduction in Ki67 expression and dramatically increased in the proportion of apoptotic cells (Figure 4L-M and Figure S8D-E). Additionally, histology analysis with HE showed no histological alternations in kidney, liver, lung and heart in treated groups, compared with control group (Figure S9). Collectively, these data demonstrated that OICR-9429 was a promising anti-tumor drug, and it combined with cisplatin might represent as a novel strategy in the treatment of PCa by improving the therapeutic outcome and reducing the dose of cisplatin.

### The target genes of WDR5 and OICR-9429 are identified in prostate cancer

**Figure 5:**
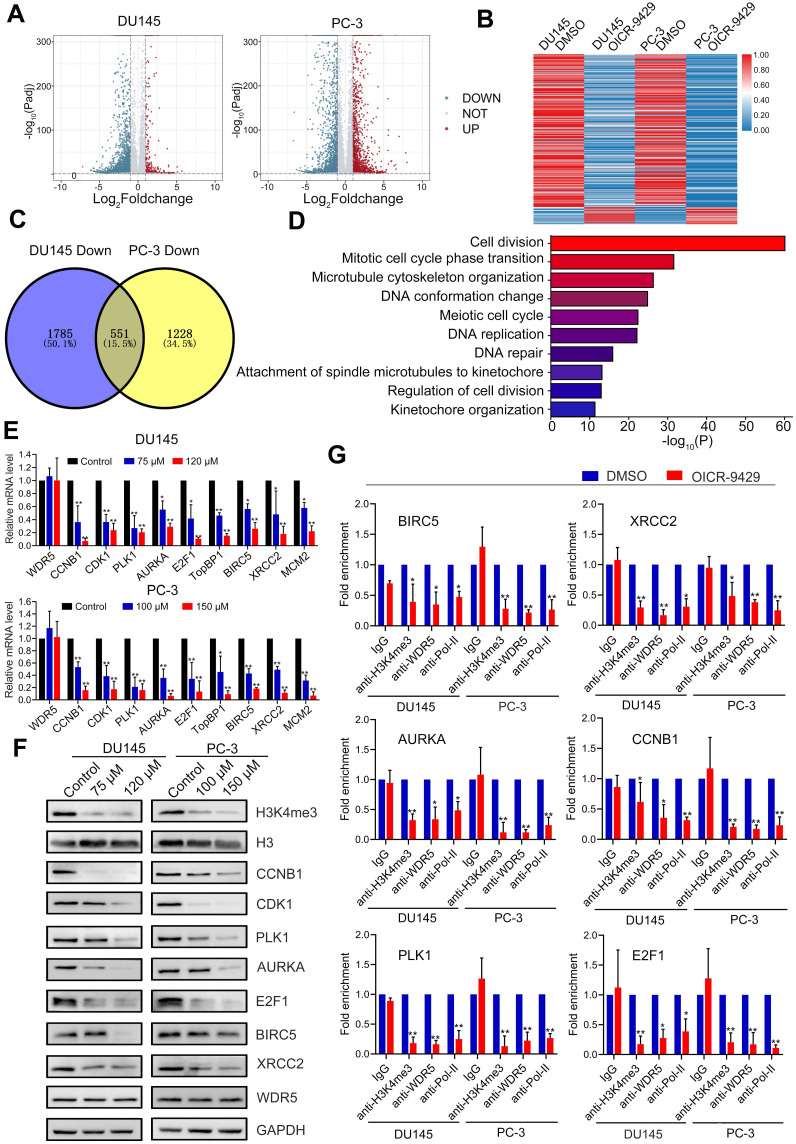
** The target genes of WDR5 are identified in PCa cells. A**. Volcano plots displaying the genes expression change in DU145 (left) and PC-3 (right) regulated by OICR-9429, compared with DMSO. Differential expression values are plotted against p-value; Red dots represent the up-regulated genes and blue dots represent down-regulated genes. **B**. A heatmap representing mRNA levels in the DU145 (left) and PC-3 (right) treated with OICR-9429 or DMSO for 48 h. Each column indicates the indicated sample, and each row represents one mRNA. Red and blue colors represent high and low expression, respectively. **C**. The Venn diagram exhibiting the number of down-regulated genes in DU145 (left) and PC-3 (right) treated with OICR-9429. 1785 genes were down-regulated in DU145 and 1228 genes were down-regulated in PC-3, 551genes were down-regulated in both DU145 and PC-3. **D**. Gene ontology (GO) analysis identifying the enrichment of the biological process. **E, F**. Validation of candidate down-regulated genes by qRT-PCR (E) and Western blotting (F) in DU145 and PC-3. GAPDH and H3 were used as internal controls. **G**. ChIP analysis of IgG, H3K4me3, WDR5 and RNA polymerase-II status at candidate WDR5 target genes in DU145 and PC-3 cells, treated with OICR-9429 or DMSO. The values are normalized to input and presented as the means ± SD. *p < 0.05; **p < 0.01.

To further clarify the mechanism of WDR5-mediated epigenetic modification in PCa, we performed transcriptomic sequencing of DU145 and PC-3 cells treated with or without OICR-9429. There were 611 genes both differently expressed in OICR-9429 treated DU145 and PC-3 cells (Figure 5A-C). Given the transcriptional activated role of MLL1-WDR5 complex, we next focus on 551 genes, which down-regulated in both PCa cells. Gene ontology (GO) analysis demonstrated that the differentially expressed genes were mainly involved in cell cycle, DNA repair and apoptosis (Figure 5D). Then, qRT-PCR analysis was performed to validate the expression of target genes from RNA-seq data. As shown in Figure 5E, cell cycle related genes, such as CDK1, PLK1, CCNB1, AURKA and E2F1, apoptosis and DNA repair related genes, such as TopBP1, MCM2, BIRC5 and XRCC2, were significantly down-regulated in OICR-9429 treating cells. Consistently, these genes were also down-regulated after knockdown of WDR5 with two siRNAs (Figure S10A). Importantly, TCGA data also shown that WDR5 were significantly positive correlated with these genes in PCa (Figure S11). Furthermore, western blotting analysis demonstrated that both OICR-9429 and knockdown WDR5 could decrease the protein expression of CDK1, PLK1, CCNB1, AURKA, E2F1, BIRC5, and XRCC2, but not TopBP1 and MCM2 (Figure 5F and Figure S10B).

Accordingly, WDR5 is both a component of MLL complex and a critical co-factor of c-Myc. Both MLL complex-mediated H3K4 methylation and c-Myc could drive tumorigenesis by regulating genes expression 15, 23. To explore whether the target genes of WDR5 were co-regulated by MLL complex and/or c-MYC, respectively, we performed qRT-PCR. As shown in Figure S12A-B, MLL1 silencing significantly reduced the mRNA level of CDK1, PLK1, CCNB1, AURKA, E2F1, BIRC5, XRCC2, and MCM2, but not TopBP1. Consistently, similarly results were also observed after c-Myc knockdown in DU145 and PC-3 cells (Figure S12C-D). However, OICR-9429 did not change the expression of both MLL1 and c-Myc in PCa cells, suggesting OICR-9429 may play pharmacological actions by blocking the interaction of WDR5 with MLL1 and c-Myc (Figure S13). Taken together, these data demonstrated that the multifunction of WDR5 might be dependent on MLL complex and c-Myc.

### Targeting WDR5 inhibits genes transcription by blocking H3K4 trimethythlation

Since the most prominent role of WDR5 is the catalytic activity of MLL1 that catalyze H3K4 methylation and H3K4me3 exhibit significant association with transcriptional activation 24, we assumed that both WDR5 knockdown and OICR-9429 suppressed the transcription of target genes directly by blocking H3K4 trimethylation. According to previous study, one of WDR5 binding-motif was CANNTG and we found that all above target genes had at least one CANNTG distributing around the TSS within 500 bps. To confirm this, we conducted chromatin immunoprecipitation (ChIP) assays in WDR5 knockdown or OICR-9429-treated and corresponding control PCa cells. As shown in Figure 5G, the enrichments of H3K4me3, WDR5 and RNA polymerase-II on the promoter regions of AURKA, BIRC5, CCNB1, E2F1, PLK1, and XRCC2 were decreased in OICR-9429-treated cells, compared with control cells. Similarly, WDR5 knockdown showed the same change of H3K4me3, WDR5 and RNA polymerase-II on the promoter regions of target genes (Figure S10C). However, the negative control and CDK1 were no obvious change (Figure S10D-E). In addition, both OICR-9429 and WDR5 knockdown reduced the binding affinities for interactions of c-Myc with promoters of target these genes (Figure S14A-B). These results demonstrated that OICR-9429 decreased cell cycle, apoptosis and DNA repair related genes by blocking the WDR5-mediated H3K4 trimethylation and disturbing the interaction of WDR5 and c-Myc with chromatin.

### Both OICR-9429 and WDR5 knockdown suppresses immune evasion by decreasing PD-L1 expression in prostate cancer

**Figure 6:**
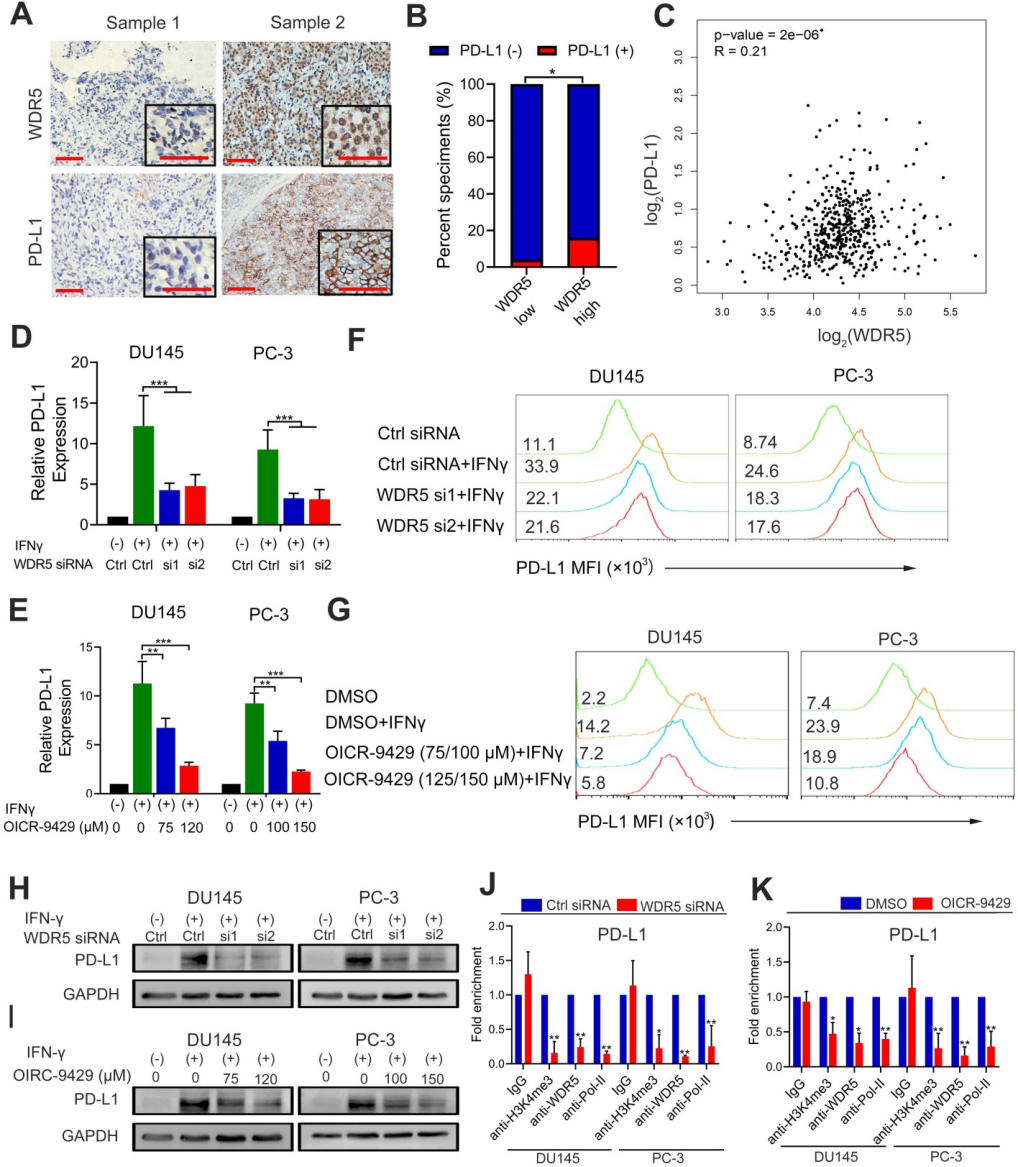
** WDR5 knockdown or OICR-9429 reduces the IFN-γ induced PD-L1 expression. A**. The representative IHC images of WDR5 and PD-L1 in PCa tissues. Scale bars: red, 50 µm. **B**. Expression difference of PD-L1 in WDR5 high expression and WDR5 low expression tissues. **C**. Pearson correlations between the expression of WDR5 and PD-L1 in TCGA cohort. **D, E**. The qPCR analysis showing that knockdown WDR5 (D) or OICR-9429 (E) decreased the mRNA expression of IFNγ-induced PD-L1 in DU145 and PC-3 cells. **F, G**. FACS staining showing that knockdown WDR5 (F) or OICR-9429 (G) reduced the expression of IFNγ-induced PD-L1 in DU145 and PC-3 cells. **H, I**. Western blotting showing that knockdown WDR5 (H) or OICR-9429 (I) reduced protein level of IFNγ-induced PD-L1 in DU145 and PC-3 cells. GAPDH was used as a loading control. Error bars indicate the standard deviations of three independent experiments. **J.** ChIP analysis of IgG, H3K4me3, WDR5 and RNA polymerase-II status at promoter of PD-L1 in DU145 and PC-3 cells, transfected with WDR5 or Ctrl siRNA. **K**. ChIP analysis of IgG, H3K4me3, WDR5 and RNA polymerase-II status at promoter of PD-L1 in DU145 and PC-3 cells, treated with OICR-9429 or DMSO. * *p* < 0.05, **p < 0.01.

Previous studies found that epigenetic modifications were involved in the formation of immunosuppressive microenvironment 25. To investigate whether WDR5 was involved in the immunomodulatory in prostate cancer, we firstly evaluated the correlation between WDR5 and PD-L1 by immunochemistry. The results demonstrated that the positive rate of PD-L1 was significantly higher in WDR5 overexpression samples (Figure 6A-B). Furthermore, the TCGA database also demonstrated that there was positive correlation between WDR5 and PD-L1 in mRNA level (Figure 6C). To investigate whether WDR5 contribute to tumor PD-L1 expression, qRT-PCR, western blotting and flow cytometry were performed. Interestingly, both PD-L1 mRNA and protein level induced by IFN-γ was significantly abrogated by WDR5 knockdown. Similarly, MLL1 silencing also reduced the mRNA level of PD-L1 in both PCa cells (Figure S15A). However, c-Myc silencing showed no significant change of PD-L1 mRNA (Figure S15B). Moreover, we observed that treatment with OICR-9429 reduced the IFN-γ-induced PD-L1 expression in a concentration-dependent manner in PCa cells (Figure 6D-I). To further explore the mechanism by which OICR-9429 and WDR5 knockdown reduced the IFN-γ-induced PD-L1 expression, we performed ChIP assay and found that both OICR-9429 and WDR5 knockdown significantly downregulated the occupancy of H3K4me3 or WDR5, but not c-MYC on the promoter of PD-L1, compared with control cells, respectively (Figure 6J-K and Figure S14A-B). These results suggested that WDR5 is essential for PD-L1 transcription in PCa, while OICR-9429 could block this process.

## Discussion

Patients with advanced PCa have an unusually poor prognosis and low response to chemotherapy and immune checkpoint inhibitors. To improve treatment and the survival rate of patients with PCa, there is an urgent need to reveal the mechanisms by which epigenetic regulators and aberrant pathways regulate oncogenic signaling and/or to discover potential markers and targets useful in PCa. In this study, we identified dysregulated expression of the histone methylation moderators WDR5 in advanced PCa and demonstrated that WDR5-mediated PCa promotion occurred via H3K4me3 and c-Myc-dependent activation of serial oncogenes.

In recent years, histone methylation moderators have increasingly broadened our insight in tumor progression. H3K27 and H3K4 are the mainly lysine methylation site and their moderators exhibit tumor-promoting role in PCa. EZH2 is the prominent subunit of the Polycomb repressive complex 2 (PRC2), a methyltransferase responsible for the all H3K27 methylation 26. Previous studies have demonstrated the EZH2 predicts poor prognosis and is involved in tumor progression in PCa, which was also confirmed in present study 27. Here, our results identified that WDR5, a subunit of SET/MLL complex responsible for H3K4 methylation, was also overexpressed and significantly associated with advanced clinicopathological characteristics and poor prognosis in PCa from multicenter cohorts. Importantly, WDR5 was an independent prognostic factor for both PFS and OS in PCa. These results attest to the contribution of WDR5 to PCa promotion and the potential usage of WDR5 as a marker in predicting malignant behavior in PCa patients.

Increasing evidences demonstrated that WDR5 displays an oncogenic role in tumor progression, including proliferation, metastasis and chemoresistance 16, 17. The previous funding indicated that WDR5 involved in the metastasis and castration resistance of PCa cells 16, 28. However, its biological role in advanced PCa remains largely unknown. In present study, we found that knockdown WDR5 suppressed the proliferation via regulating cell cycle, and induced the apoptosis and increased the chemosensitivity of PCa cells to cisplatin by weakening DNA damage respire (DDR). The finding was similar to our previous data in bladder cancer 17. Collectively, those results suggested that WDR5 was a potential target for improving the chemosensitivity in PCa.

Targeting the gene by siRNA *in vivo* is operose and immature currently. However, the small molecule compound OICR-9429 is optimized for high selectivity and potency against WDR5. OICR-9429 could competitively bind to WIN and WBM site of WDR5 to block the interaction WDR5 with corresponding partners, and consequently reduce transcription of target genes 13. OICR-9429 has displayed anti-tumor effect by inhibiting the proliferation in leukemia and pancreatic cancer. In present study, we firstly demonstrated that OICR-9429 suppressed the proliferation of PCa cells via selectively arresting cells at G1/S phase, and inducing the apoptosis of PCa cell. Moreover, our funding indicated that OICR-9429 could significantly increase the cisplatin sensitivity by blocking the DDR in PCa cells. Those data highlight an optimal strategy to target PCa by OICR-9429 via inhibiting PCa cell viability as well as increasing chemosensitivity to cisplatin. However, the target genes of OICR-9429 in PCa are unclear, and it is an important point for further pre-clinical and clinical trials. To address this, we firstly performed RNA-sequencing and found 551 genes were down-regulated in both DU145 and PC-3 cells. Moreover, GO analysis further demonstrated most of down-regulated genes are involved in cell division, cell cycle phase transition and DNA repair. Interestingly, qPCR, western blotting verification indicated that AURKA, BIRC5, CCNB1, E2F1, PLK1 and XRCC2 were the direct targets of OICR-9429. Similar results were also found in knockdown WDR5, MLL1 or c-Myc by siRNA. Moreover, both OICR-9429 and WDR5 knockdown significantly reduced the level of H3K4me3 and recruitment of c-Myc on these promoters of target genes. Accordingly, H3K4 methylation events, which marked transcriptionally-active chromatin, correlated tightly with c-Myc binding across genome and even called as “strict prerequisite” for target gene recognition by c-Myc 29. Considering the direct interaction of WDR5 with MLL complex and c-Myc via two different binding sites 30, it is reasonable that WDR5 formed a protein complex with c-Myc and MLL1 at the promoter of target genes, leading to an amplifying transcription. Thus, OICR-9429 suppressed the expression of target genes by blocking the formation of mentioned protein complex. Of note, all of the OICR-9429 directly targeted genes exhibit multiple oncological events. Among these genes, AURKA, CCNB1, E2F1 and PLK1 are well known in promoting different cell cycle phase transition. It is proven that BIRC5 and XRCC2 regulate anti-apoptosis and DNA repair. Surprisingly, all six genes above mentioned could regulate DDR in different steps. AURKA could activate ATM/Chk2 signal and negatively regulated P53 expression to repair DNA damage 31, 32. CCNB1/CDK1 functions to deliver signals to mitochondria to boost ATP generation for DNA repair and cell survival BIRC5 could protect chromosome stability independently from P53 33. XRCC2 participates in homologous recombination repair of damaged DNA 34. E2F1 plays a role in Chk1-mediated DNA damage checkpoint control 35. While, PLK1 plays a vital role during recovery from G2 DNA damage checkpoint by targeting multiple factors downstream of ATM and ATR 36. Therefore, WDR5 is the upstream of these cell cycle and DDR related oncogenes. Targeting WDR5 by OICR-9429 is a multi-potency and promoting anticancer therapy in PCa, which increase cisplatin sensitivity through disrupt multiple steps of DDR.

Immune checkpoint inhibitors of PD-1/PD-L1 show promising therapy response in some cancers, but they are less effective in PCa. It's vital to clarify the expression regulators of PD-L1. It had been reported that both EZH2 inhibitor and BRD4 inhibitor could reduce the IFN-γ induced PD-L1 expression in PCa cells 25, 37, suggesting that epigenetic regulator inhibitors could reverse the dilemma of ICIs. In present study, we found that WDR5 was positively associated with PD-L1 in both mRNA and protein levels in TCGA and our center cohorts. Interestingly, our results showed that both targeting WDR5 by siRNA and OICR-9429 could obviously reduce the IFN-γ induced PD-L1 transcription and expression. In addition, knockdown MLL1 but not c-Myc could also reduce the IFN-γ induced PD-L1 transcription. Furthermore, ChIP assays demonstrated that OICR-9429 could down-regulated the promoter H3K4me3 and WDR5 levels of PD-L1. Collectively, these results suggested that OICR-9429 may be an alternative agent for combing immunotherapy to improve the clinical outcome since it inhibits the PD-L1 expression in tumor cancer surface and exosomes.

**Figure 7:**
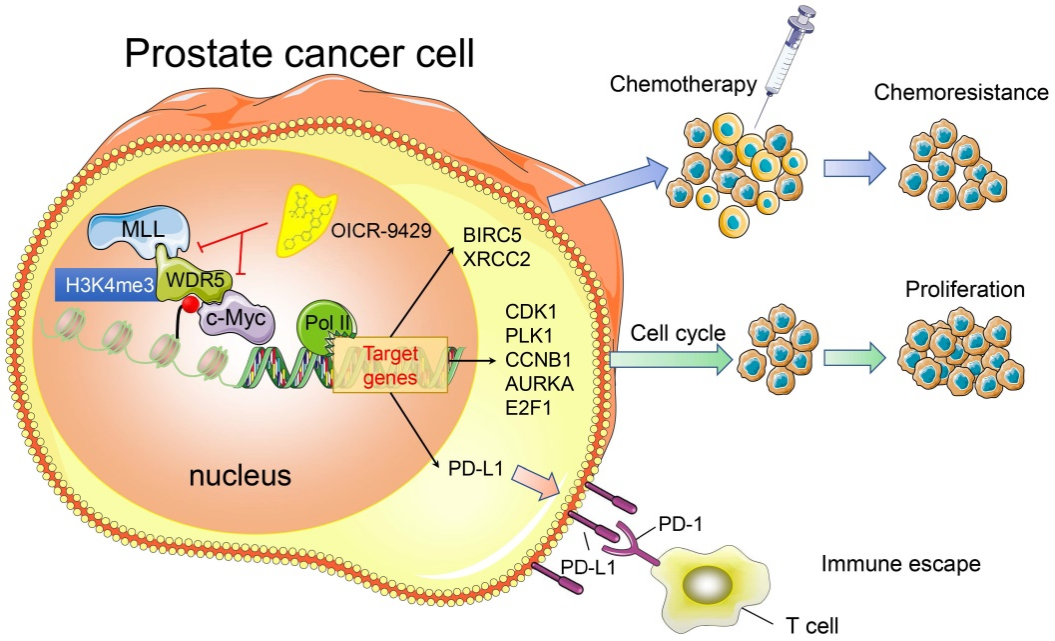
A model depicting that inhibition of WDR5 suppresses proliferation. chemoresistance and immune escape by transcriptionally.

In summary, we provide evidence that WDR5 activates cell cycle, DNA repair, anti-apoptosis and PD-L1 signaling, thereby promoting PCa progression. The use of OICR-9429 to block WDR5-mediated functions reverses oncogenic characteristics in PCa (Figure 7). Importantly, our studies contribute to a better understanding of how WDR5 participates in the regulation of translational modifications, and OICR-9429 is a multi-potency targeted therapy, which combined with cisplatin and/or ICIs may be promising therapeutic strategies for advanced PCa.

## Materials and methods

### Clinical samples

With the approval from the Institutional Ethical Boards of Sun Yat-sen Memorial Hospital (SYMH), we obtained 136 formalin-fixed, paraffin-embedded (FFPE) primary PCa specimens and 26 normal adjacent prostate tissues from SYSUCC (termed Cohort1) between January 2005 and December 2013, and 126 primary PCa samples and 19 normal adjacent prostate tissues from SYMH (termed Cohort 2) between January 2008 and December 2013. Patients from both cohorts signed informed consents. Two senior pathologists were assigned to independently confirm the Gleason score, T stage and N status with the H&E slides. The basic clinicopathological information of both cohorts was listed in Table S3.

### The TCGA data mining

Patients' clinical profiles in the TCGA prostate adenocarcinoma cohort were obtained from TCGA https://cancergenome.nih.gov/. The basic clinicopathological features of patients are listed in Table S3. In line with previous study, the TCGA prostate adenocarcinoma cohort containing 374 patients was used for the analysis and patients without detailed clinical data were excluded 33. Thus, cases with complete clinicopathological data and follow-up information were included for the univariate and multivariate analyses.

### Immunohistochemistry (IHC) and score

The immunohistochemical staining was carried out on FFPE tissue as previous described 38, 39. The staining intensity was scored as negative (0), weak (1), medium (2) and strong (3) (Figure S16). For evaluating the expression of WDR5, the staining extent was scored percentage of immunoreactive tumor cells. A score ranging from 0 to 300 was calculated by multiplying the staining intensity with corresponding staining extent and the cases were classed as low (score <85) or high (score ≥85) WDR5 expression. However, PD-L1 expression on the tumor cell surface was scored, based on the percentage of positive cells, in line with our previous study 40.

### Cell culture

Human normal prostate cell WPMY-1 and prostate cancer cell DU145 and PC-3 were purchased from American Type Culture Collection (ATCC). WPMY-1 and DU145 were grown in DMEM (Gibco), whereas PC-3 was grown in RPMI 1640 (Gibco), supplemented with 10% fetal bovine serum, 1% penicillin-streptomycin at 37 °C with 5% CO_2_. Mycoplasma was tested every 2 weeks in all cells used in this study.

### RNA interference

All siRNAs, targeting WDR5 and negative control, were purchased from GenePhama (Shanghai, China), and the sequence of all siRNAs was listed in Table S4. In line with our previous study, siRNAs were transfected into cells with the final concentration of 10 μM, under assist of Lipofectamine RNAimax (Life Technologies) 41.

### Stable WDR5 Knockdown Cell Lines

The pLKO.1 TRC cloning vector (Addgene plasmid) was used to establish a short hairpin RNA (shRNA) against WDR5 (5'-GCTCAGAGGATAACCTTGT-3') or negative control (5'-TTCTCCGAACGTGTCACGT-3'). Lentivirus production and infection were consistent with our previous study 42.

### Real-time quantitative PCR (qRT-PCR)

Total RNA was extracted from cells with TRIzol (Invitrogen), following the manufacturer's introduction. Reverse transcription was performed with the PrimerScript RT-PCR kit (TaKaRa biotechnology, Dalian, China). A standard SYBR Green PCR kit (Roche) was used for quantitative RT-PCR on a LightCycler 480 system. Relative levels of mRNAs were calculated using the 2^-ΔΔCt^ method and the GAPDH was used as an internal control. The primers were listed in Table S5.

### Western blot assay and antibodies

Western blot was carried out according to our previous study 43. Primary antibodies specific to WDR5, H3, H3K4me3, AURKA, BIRC5, CCNB1, CDK1, E2F1, PLK1, XRCC2, PD-L1, MLL1, c-Myc and GAPDH were used. After incubation with primary antibodies, members were incubated with corresponding secondary antibodies (Cell Signaling Technology). Membranes were visualized using enhanced chemiluminescence.

### Cell proliferation assays

A methyl thiazolyl tetrazolium (MTT) assay was used to assess cell viability. In brief, cells were grown in 96-well culture plates, and incubated with the MTT labeling reagent for 2 h. then the medium was replaced with dimethyl sulfoxide medium. The formazan levels were determined at 570 nm.

For the colony formation assay, approximately 500 PCa cells were seeded per well in six-well plates for 10 days, subsequently, the clones were fixed with 4% paraformaldehyde and stained with crystal violet for approximately 20 min. Then, the clones were imaged and calculated with Quantity One 1-D Analysis software (Bio-Rad, Hercules, CA, USA).

For cell cycle analyses, after removal of suspended cells, DU145 or PC3 cells were harvested and fixed with 70% cold ethanol at 4 °C overnight. Then, cells were incubated with PBS containing 50 μg/mL propidium iodide (PI) and 100 μg/mL RNase A for 30 min. Date acquisition was carried out with FACS Vantage flow cytometer and analyzed with FlowJo software.

The ethynyl deoxyuridine (EdU) assays were carried out according to the manufacture's protocol (RiboBio, Guangzhou, China) and the details were described in previous study 17.

### Apoptosis analysis

Following transfected with WDR5 siRNA or control siRNA, and/or treated with agents for 48 h, the PCa cells were collected and the cell apoptosis was analyzed with Annexin V-FITC and PI (KeyGEN Biotech, Nanjing, China) staining by using a FACSCaliber BD flow cytometer. The TUNEL assay was conducted with the *In situ* Cell Death Detection Kit (Roche), in line with previous study 16.

### Comet assay

The comet assay was carried out according to the manufacture's protocol (Trevigen#4250-050) and the percent tail DNA was calculated using CometScore 2.0. At least 30 cells were analyzed and plotted using prism. The Kurskal-Wallis test was used for statistical analysis 44.

### Chemosensitivity assay and drug synergistic effect calculations

The PCa cells were treated with a series of different concentrations of OICR-9429 (0, 12.5, 25, 50,100,200,400 μM) or cisplatin (0, 0.5, 1, 1.5, 2, 2,5, 3 μg/mL) for 48 h. the cell viability was measured by MTT assay. Both the calculation of IC_50_ and the dose-response curve were completed with GraphPad Prism 8. For testing synergistic effect of OICR-9429 and cisplatin, the PCa cells were treated with each drug alone or in combination and then the cell viability was measured by MTT assay. The Combinational Index (CI) was calculated by using CalcuSyn software (Biosoft) and CI<1.0 was considered to be synergistic effect 45.

### RNA sequencing analysis

Total RNA was extracted from DU145 or PC-3 cells treated with DMSO or OICR-9429 for 36 h, respectively, using TRIzol reagent (Invitrogen). Library construction and sequencing were conducted by Annoroad Gene Technology (Beijing, China), in line with previous study 43. All primary data in RNA sequencing (RNA-seq) analysis have been uploaded to the Gene Expression Omnibus (GSE166136).

### ChIP assay

The ChIP assays were conducted according to the manufacturer's instructions (Thermo, USA) and previous described 38. In brief, after transfected WDR5 siRNA or control siRNA, or treated with OICR-9429 or DMSO for 48 h, PCa cells were cross-linked with 1% formaldehyde, followed by neutralization with glycine. Then cells were lysed, sonicated and bound with the indicated antibody (anti-WDR5, anti-H3K4me3, anti-RNA polymerase-II and IgG), at 4 °C overnight. After that, chromatin-antibody complex was further incubated with magnetic beads on ice for 2 h and collected. Finally, the beads were washed 3 times and the DNA was purified with phenol chloroform extraction and ethanol precipitation. Primers for ChIP-qPCR were listed in Table S6.

### Xenograft tumor studies

All animal procedures in present study were approved by the Sun Yat-sen University Institutional Animal Care and Use Committee and the approval number is L102042019070C. All male BALB/c nude mice were purchased from Shanghai SLAC Laboratory Animal Co., Ltd. The age of mice was four-to five-week-old. For part animal procedures of combination WDR5 knockdown with cisplatin, 3×10^6^ PC-3 cells stably transfected with sh-control or sh-WDR5 were injected subcutaneously into the flank of each mouse. After 1 week, the nude mice bearing sh-WDR5 or sh-control xenografts were subdivided randomly into two groups for treatment with cisplatin (3 mg/kg) or PBS. For animal procedures of combination OICR-9429 and cisplatin, 3×10^6^ wild type PC-3 cells were injected subcutaneously into the flank of mice and the mice bearing xenografts were randomly assigned to four groups: (1) Control group, treated with PBS per 3 days; (2) OICR-9429 group, treated with OICR-9429 at 10 mg/kg per 3 days; (3) Cisplatin group, treated with cisplatin at 3 mg/kg per 3 days; and (4) Combination group, treated with OICR-9429 and cisplatin per 3 days. The animals were treated for four weeks. The volumes of the xenografts were measured per 3 days and the tumors were dissected surgically following the euthanasia of mice. Finally, the tumors were fixed with 4% paraformaldehyde and embedded in paraffin.

### Statistical analysis

All statistical analyses were performed with or SPSS version 24.0 or GraphPad Prism 8.0 software. A two-tailed Student's test for two-group comparisons, or a two-way ANOVA test followed by Dunnett's tests for multiple group comparisons, were utilized to assess the data. Pearson's test was applied to evaluated the correlation between WDR5 expression and clinicopathological variables. Kaplan-Meier analysis with log-rank test was performed for survival rate analyses. The survival hazard ratios and 95% confidence intervals were calculated using Cox proportional hazard regression models. Data were presented as the mean ± SD. Differences at p<0.05 were considered statistically significant.

## Supplementary Material

Supplementary figures and tables.

## Abbreviations

PCa: prostate cancer
BPH: benign prostate hyperplasia
WDR5: WD repeat domain 5
PD-L1: programmed death ligand-1
CHIP: chromatin immunoprecipitation
AURKA: aurora kinase A
CCNB1: cyclin B1
E2F1: E2F transcription factor 1
PLK1: polo like kinase 1
BIRC5: baculoviral repeat containing 5
XRCC2: X-ray repair cross complementing 2
ADT: androgen deprivation therapy
CRPC: castration-resistant prostate cancer
Enz: Enzalutamide
ICI: immune checkpoint inhibitors
H3K4: histone H3 lysine 4
TSS: transcription start sites
EZH2: enhancer of zeste 2 polycomb repressive complex 2 subunit
PFS: progression free survival
OS: overall survival
MTT: methyl thiazolyl tetrazolium
CI: combination index
TopBP1: DNA topoisomerase II binding protein 1
MCM2: minichromosome maintenance complex component 2
CDK1: cyclin dependent kinase 1
DDR: DNA damage respire
ATM: ataxia-telangiectasia mutated
Chk2: checkpoint kinase 2
Chk1: checkpoint kinase 1
ATR: ataxia-telangiectasia and Rad3-related
SYSUCC: Sun Yat-sen University Cancer Center
SYMH: Sun Yat-sen University Cancer Center
FFPE: formalin-fixed, paraffin-embedded
IHC: immunochemistry
EdU: ethynyl deoxyuridine
siRNA: small interference RNA
PCR: polymerase chain reaction
qRT-PCR: quantitative real-time PCR
WB: western blotting
TCGA: the cancer genome atlas
