# Supplementary material for: WD repeat domain 5 promotes chemoresistance and Programmed Death-Ligand 1 expression in prostate cancer
Source: Theranostics. 2021 Mar 4;11(10):4809–24. doi: 10.7150/thno.55814 (PMC7978315; doi:10.7150/thno.55814)
Supplement: Supplementary file 1 — Supplementary figures and tables. [file thnov11p4809s1.pdf]

WD repeat domain 5 promotes chemoresistance and Programmed Death-Ligand 1 expression in prostate cancer

Authors: Qianghua Zhou[1,2, #], Xu Chen[1,2, #, *], Haixia He[3,4, #], Shengmeng Peng[1,2], Yangjie Zhang[1,2], Jingtong Zhang[1,2], Liang Cheng[1,2], Sen Liu[1,2], Ming Huang[1,2], RuihuiXie[1,2], Tianxin Lin[1,2, 5, *], Jian Huang[1,2, *]

Affiliations

1. Department of Urology, Sun Yat-sen Memorial Hospital, Sun Yat-sen University, Guangzhou 510120, China.
2. Guangdong Provincial Key Laboratory of Malignant Tumor Epigenetics and Gene Regulation, Sun Yat-Sen Memorial Hospital, Sun Yat-Sen University, Guangzhou 510120, China.
3. State Key Laboratory of Oncology in South China & Collaborative Innovation Center of Cancer Medicine, Sun Yat-sen University Cancer Center, Guangzhou, 510060, China.
4. Department of Medical Oncology, Sun Yat-sen University Cancer Center, Guangzhou, 510060, China
5. Department of Urology, The Affiliated Kashi Hospital, Sun Yat-sen University, Kashi, China

[#] The first 3 authors contributed equally in this paper.

[*]**Corresponding Author:**

Xu Chen, Tianxin Lin and Jian Huang, Department of Urology, Sun Yat-sen Memorial Hospital, 107th Yanjiangxi Road, Guangzhou, China. E-mail: chenx457@mail.sysu.edu.cn, lintx@mail.sysu.edu.cn, huangj8@mail.sysu.edu.cn

Figure legends

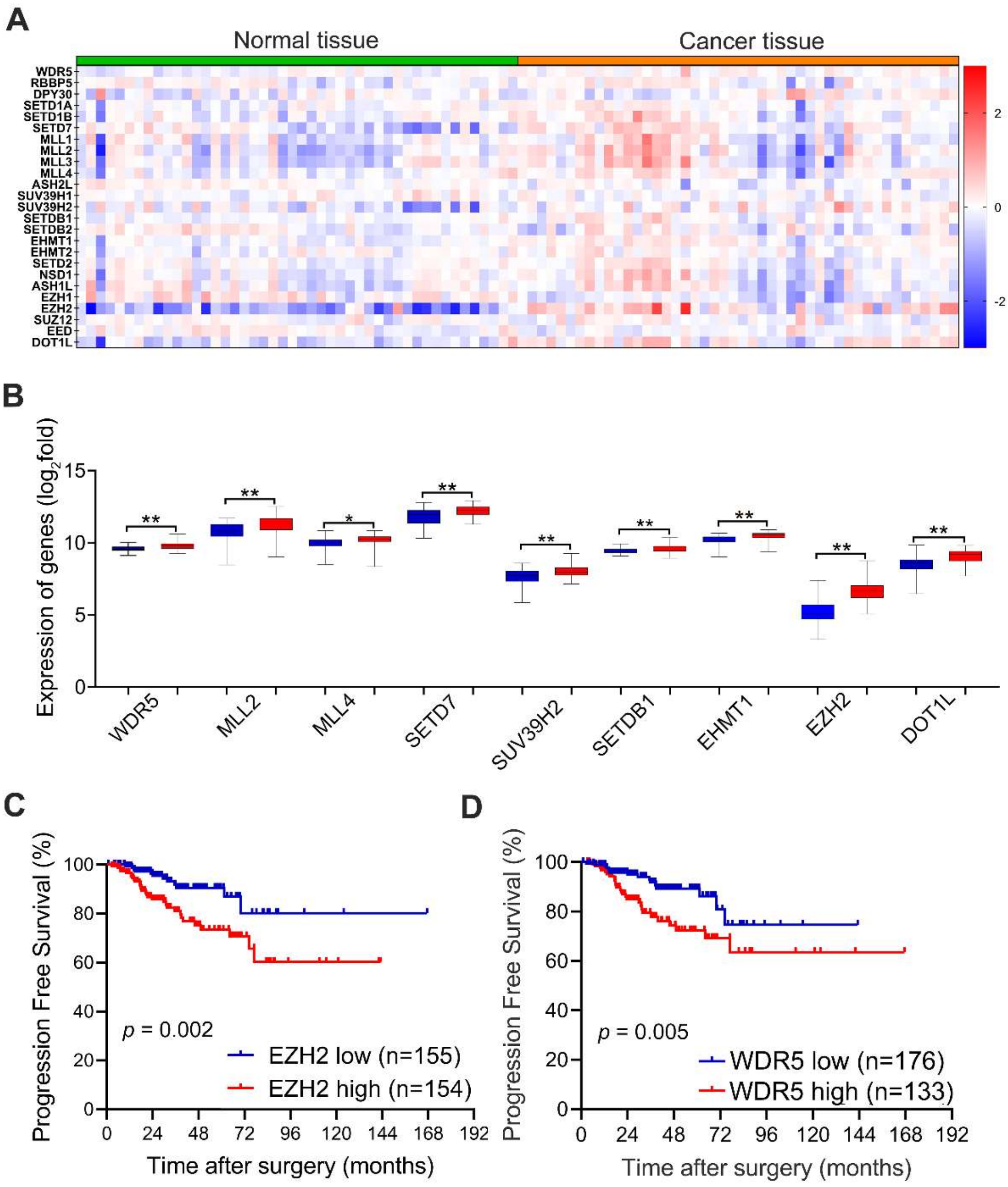

Figure S1. An integrated analysis of the histone methylation modification regulators expression in TCGA data. A. A heat map showing the expression of 25 histone methylation modifiers in adjacent normal (left) and PCa (right) tissues. **B**. 9 histone methylation modifiers were overexpressed in PCa, compared with adjacent normal tissues. **C, D**. Kaplan-Meier curves for Progression free survival of PCa patients with high vs. low expression of EZH2 (C) or WDR5 (D) in TCGA Cohort. *$p < 0.05$, **$p < 0.01$

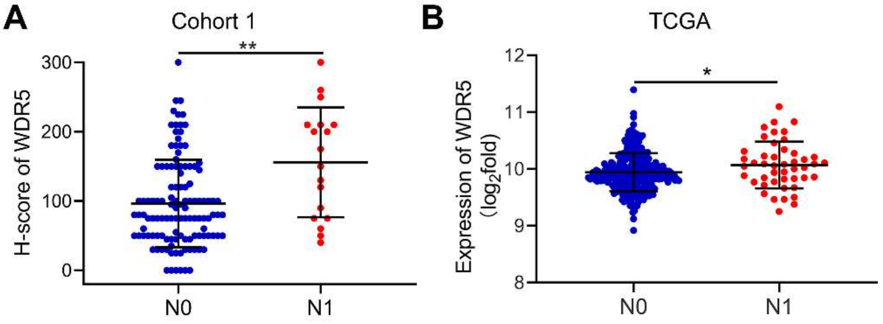

Figure S2. The expression of WDR5 in PCa tissues with or without LN metastasis in Cohort 1 (A) and TCGA cohort (B).

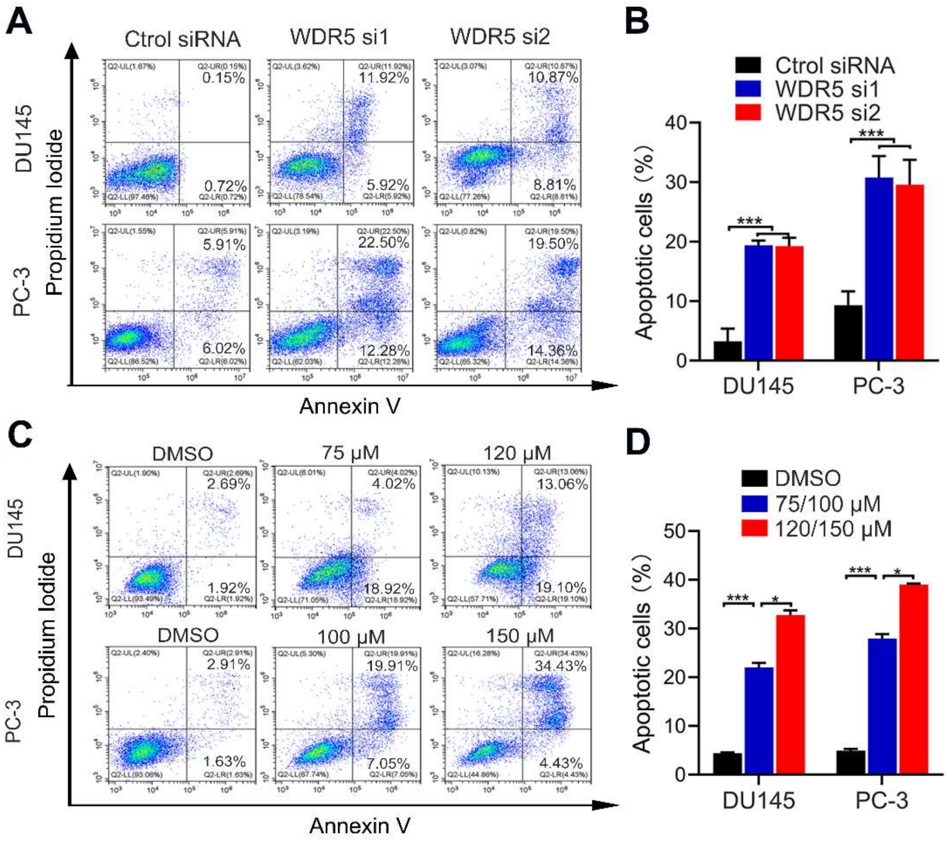

Figure S3. Knockdown WDR5 or OICR-9429 increase the apoptotic proportion in PCa cells. A, B. Representative images (A) and quantification (B) of cell apoptosis in DU145 and PC-3 cells transfected with WDR5 or Ctrl siRNA, analyzed by flow cytometry analysis. **C, D**. Representative images (C) and quantification (D) of cell apoptosis in DU145 and PC-3 cells treated with OICR-9429 or DMSO, analyzed by flow cytometry analysis. The error bars represent standard deviations of three independent experiments. *$p < 0.05$, **$p < 0.01$

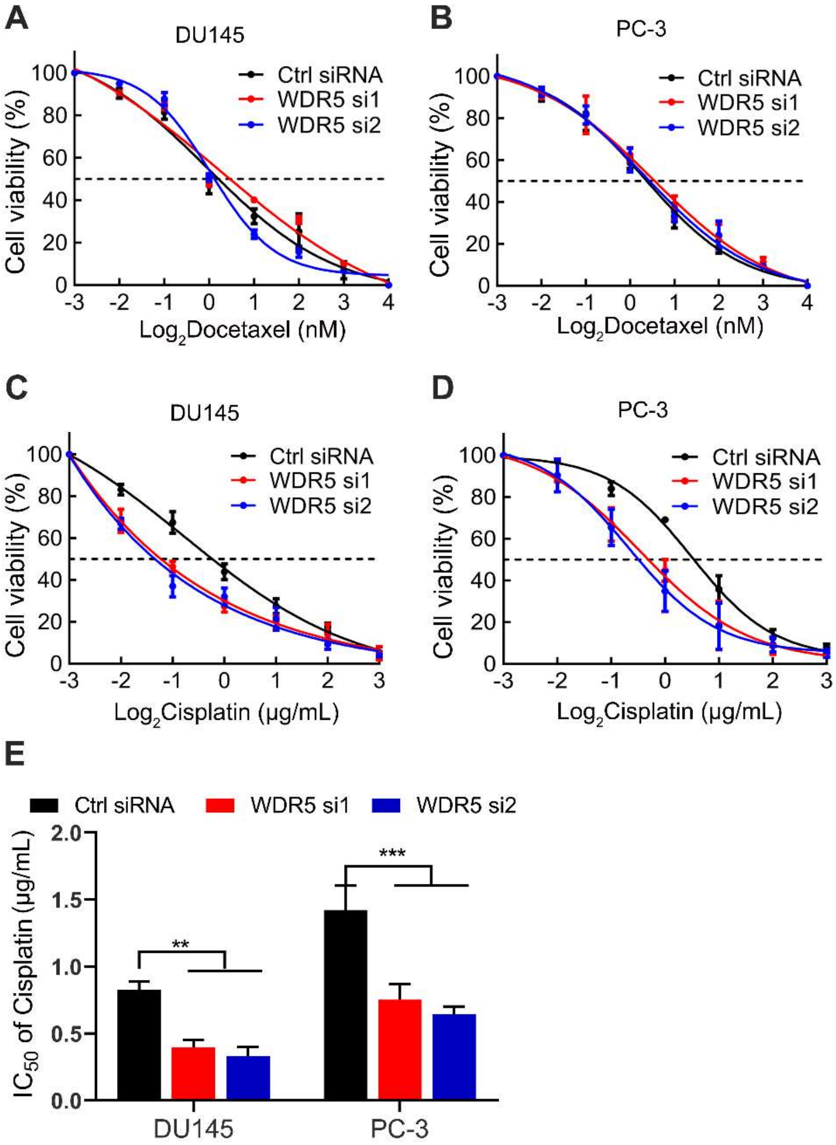

Figure S4. Knockdown WDR5 increases the chemosensitivity to cisplatin but not docetaxel in PCa cells. A-D. The cell viability of knockdown WDR5 by siRNA combined with docetaxel (A-B) or cisplatin (C-D) in DU145 and PC-3 cells by MTT assay. **E**. The quantification of IC_{50} of cisplatin in DU145 and PC-3 cells transfected with WDR5 or Ctrl siRNA. The error bars represent standard deviations of three independent experiments. $^{**}p < 0.01$.

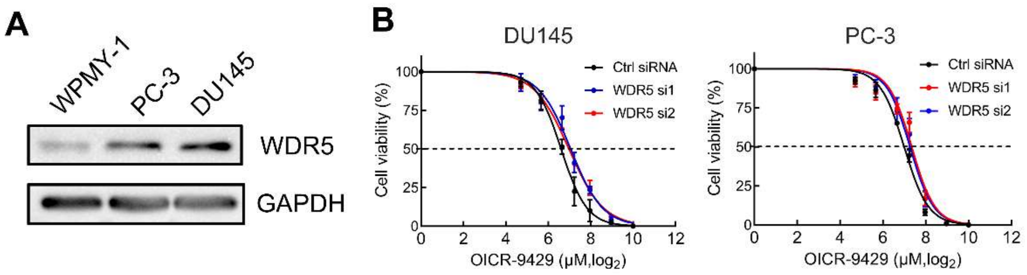

Figure S5. A. The protein level of WDR5 in WPMY-1, PC-3 and DU-145 cells. B. Knockdown of WDR5 reduced OICR-9429 sensitivity in DU145 and PC-3 cells.

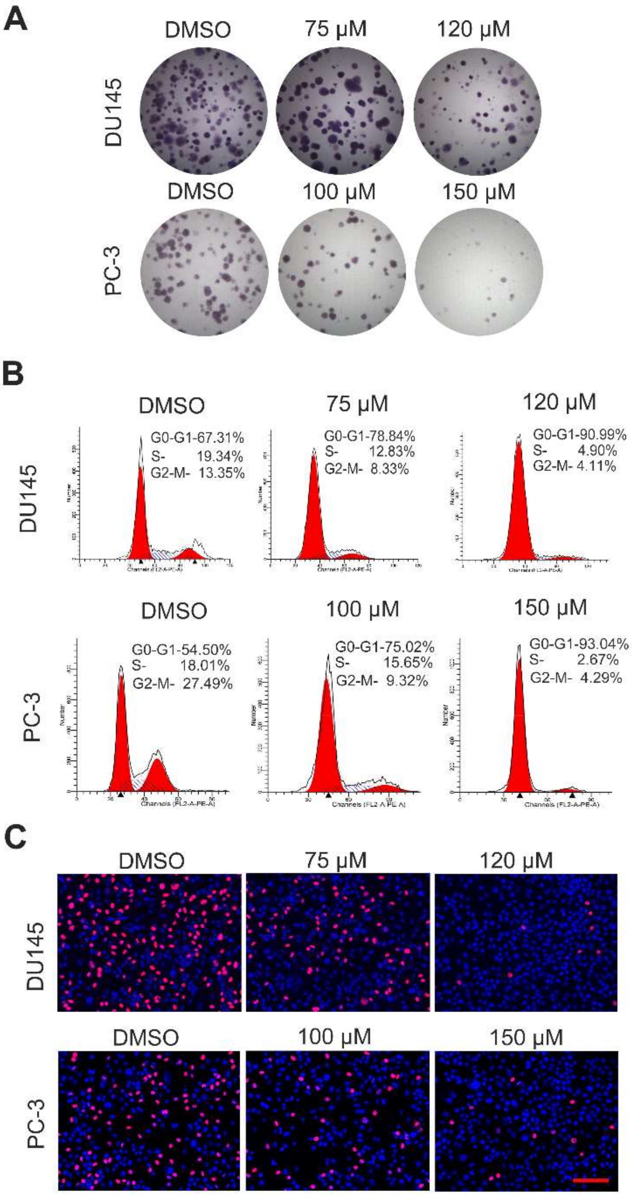

Figure S6. OICR-9429 inhibits proliferation of PCa cells in *vitro*. A. The images of formation assay of DU145 and PC-3 cells treated with OICR-9429 or DMSO. **B**. Representative images of cell cycle in DU145 and PC-3 cells treated with OICR-9429 or DMSO, analyzed by flow cytometry analysis. **C**. The images of EdU assay of DU145 and PC-3 transfected with WDR5 or Ctrl siRNA. Scale bars: red, 50 μm.

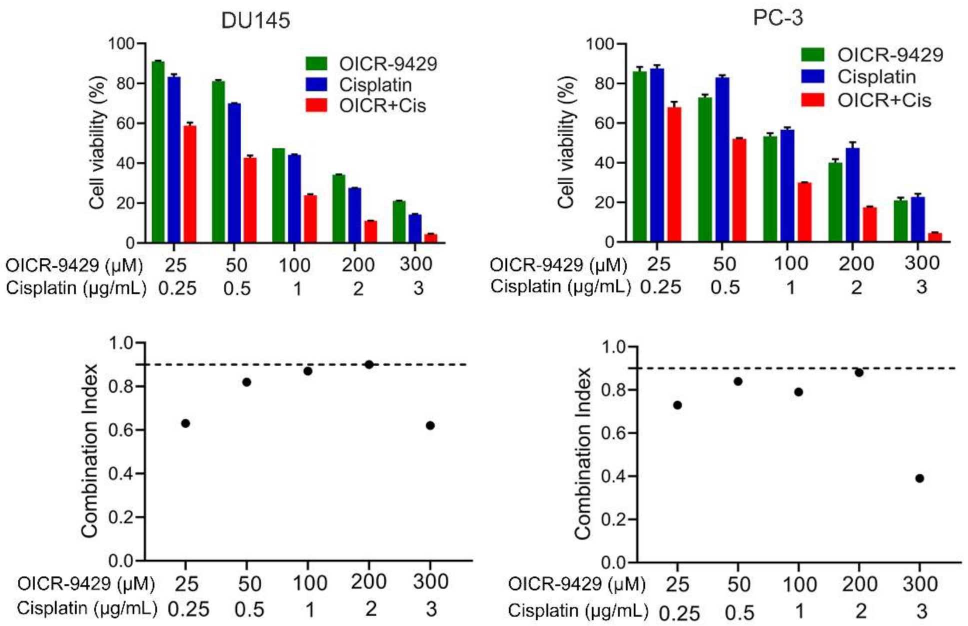

Figure S7. Synergistic effects of OICR-9429 and Cisplatin against PCa cells. DU145 and PC-3 cells treated with OICR-9429 and Cisplatin alone or in combination at indicated concentrations for 48 h. Cell viabilities were measured and normalized to DMSO control values (top). CI was calculated by using CalcuSyn software (bottom). CI less than 0.9 demonstrates synergy between two drugs.

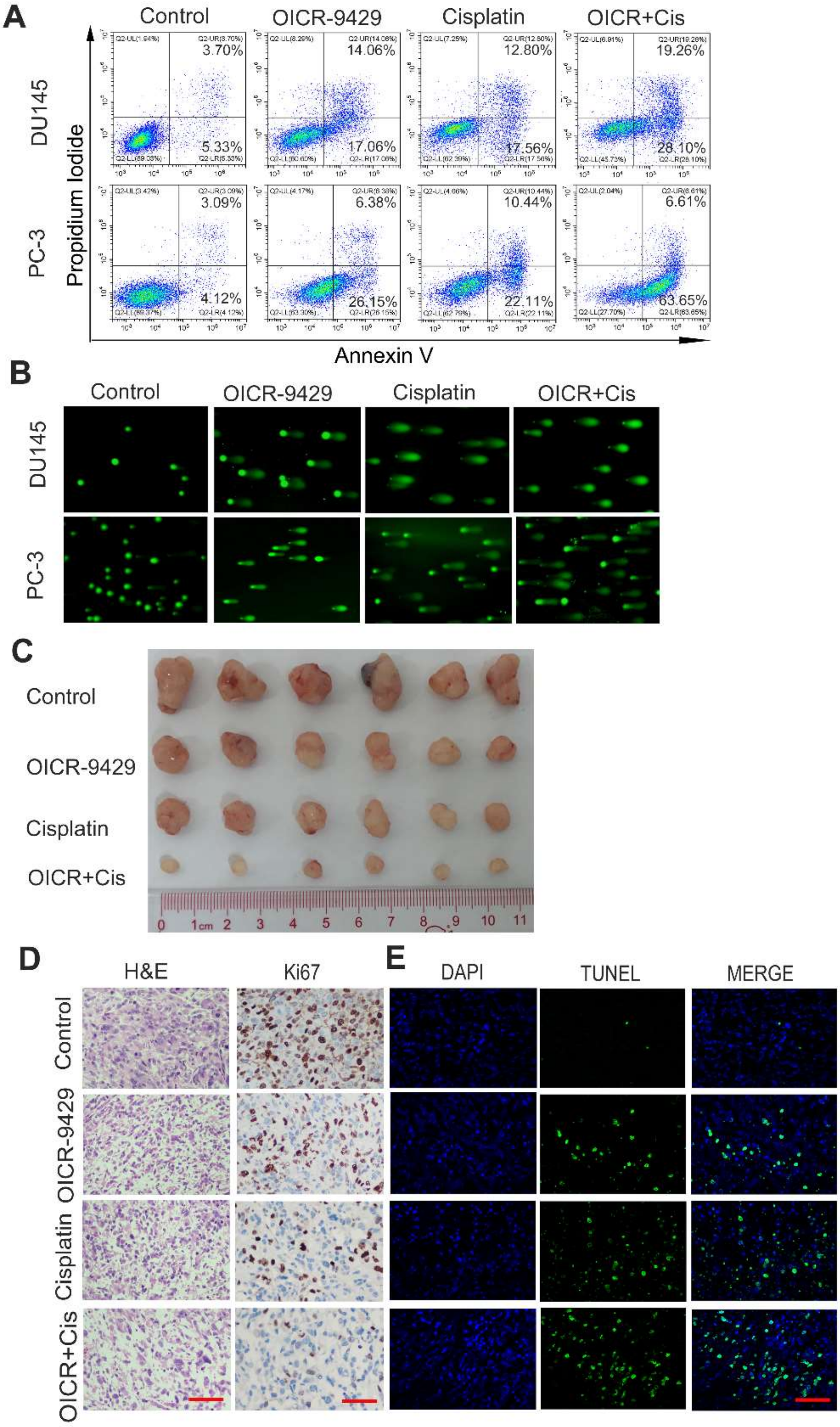

Figure S8. OICR-9429 enhances the efficacy of cisplatin in PCa cells in v*itro* and *in vivo*.

A. Representative images of cell apoptosis in the indicated cells treated with OICR-9429, Cisplatin or a combination of both for 48 h. **B**. Representative images of Comet assay in PCa cells treated with OICR-9429, Cisplatin or a combination of both for 48 h. **C**. Images of tumors

in indicated groups. **D**. Representative images of Ki67 expression in tumors of indicated groups, examined by IHC. **E**. Images of apoptosis in tumors of indicated groups, detected by TUNEL assay. The scale bars in IHC and TUNEL images represent 50 μm.

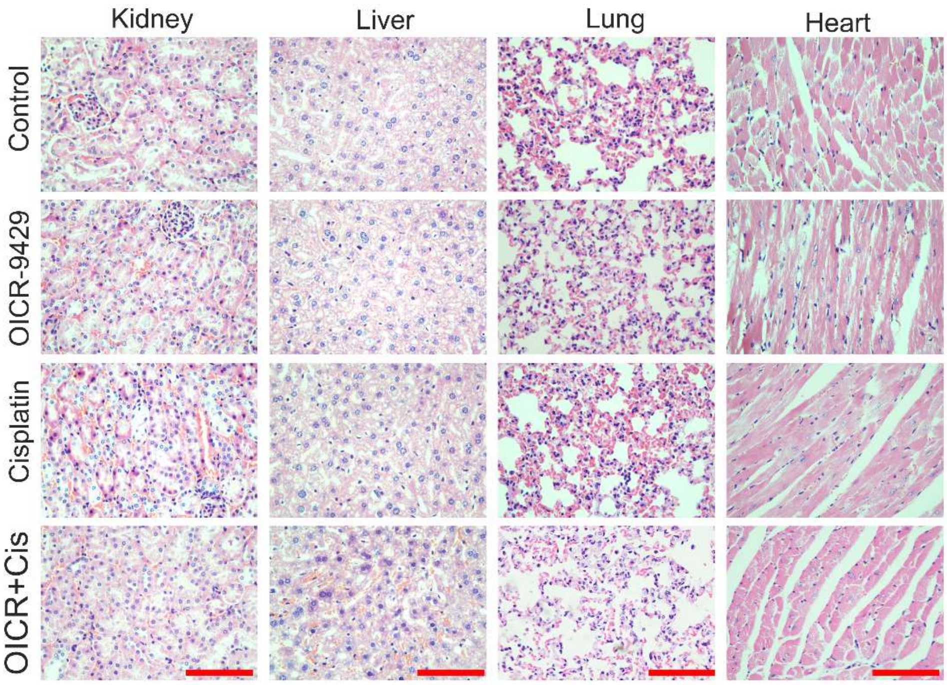

Figure S9. H&E staining of kidney, liver, lung, and heart from the mice in indicated groups. Scale bars: red, 50 μm.

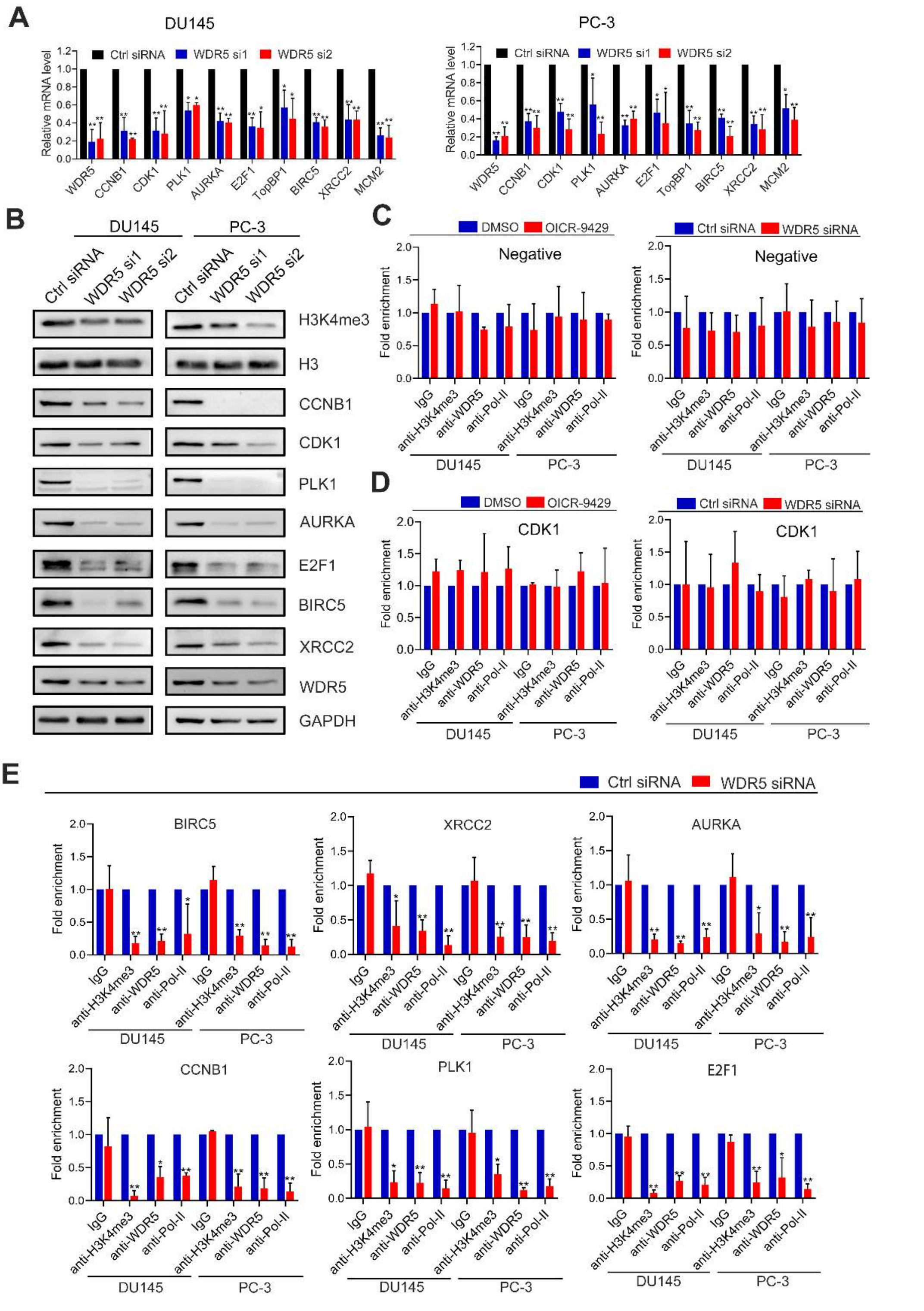

Figure S10. The target genes of WDR5 are identified in PCa cells. A, B. Validation of candidate down-regulated genes by qRT-PCR (A) and Western blotting (B) in DU145 and PC-3. GAPDH and H3 were used as internal controls. **C-E**. ChIP analysis of IgG, WDR5, H3K4me3, and RNA polymerase-II status of candidate WDR5 target genes in DU145 and PC-3 cells,

transfected with WDR5 or Ctrl siRNA. The values are normalized to input and presented as the means ± SD. The error bars represent standard deviations of three independent experiments. *$p < 0.05$, **$p < 0.01$.

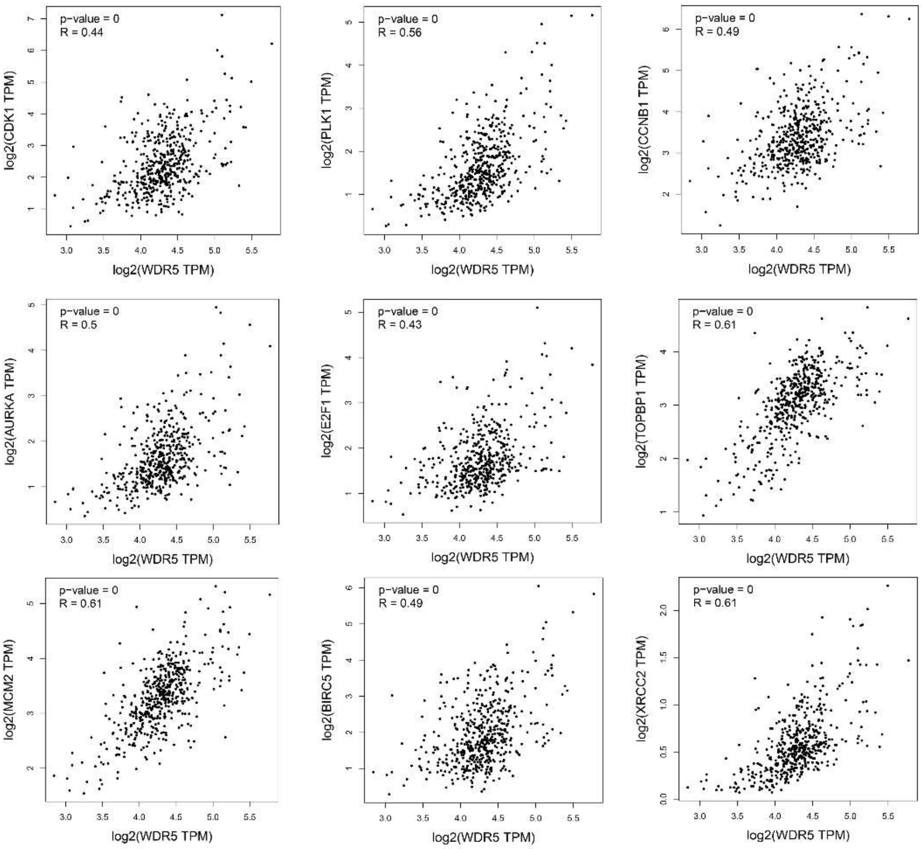

Figure S11. Pearson correlations between the expression of WDR5 and CDK1, PLK1, CCNB1, AURKA, E2F1, TopBP1, MCM2, BIRC5 and XRCC2 in TCGA cohort.

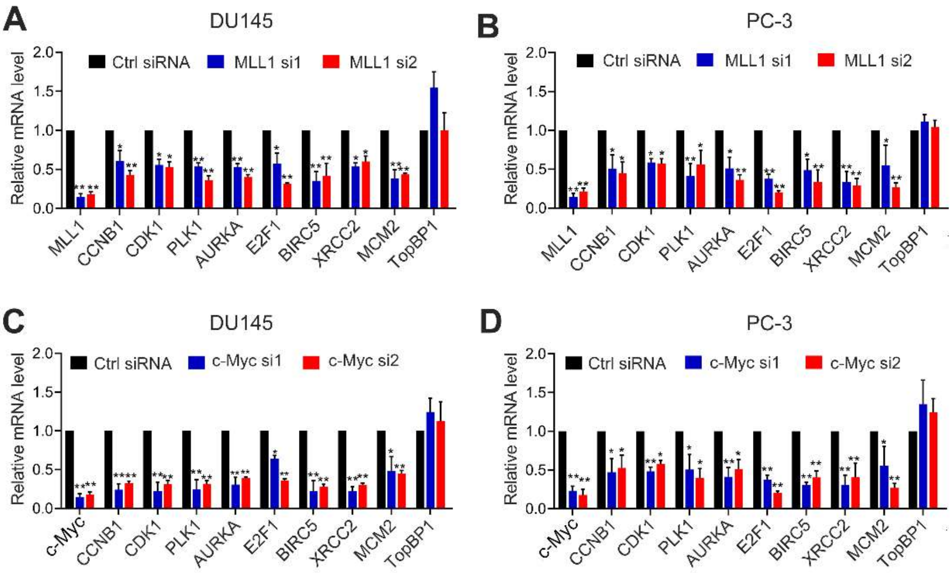

Figure S12. The WDR5 target genes are regulated by MLL1 and c-Myc. A, B. MLL1 silencing down-regulated the mRNA level of WDR5 target genes in DU145 (A) and PC-3 (B) cells. **C, D**. c-Myc silencing down-regulated the mRNA level of WDR5 target genes in DU145 (C) and PC-3 (D) cells. The values are normalized to input and presented as the means ± SD. The error bars represent standard deviations of three independent experiments. *$p < 0.05$, **$p < 0.01$.

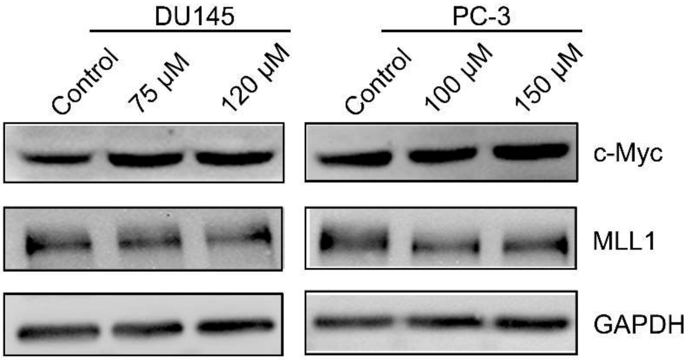

Figure S13. OICR-9429 did not change the expression of both c-Myc and MLL1 in DU145 and PC-3 cells.

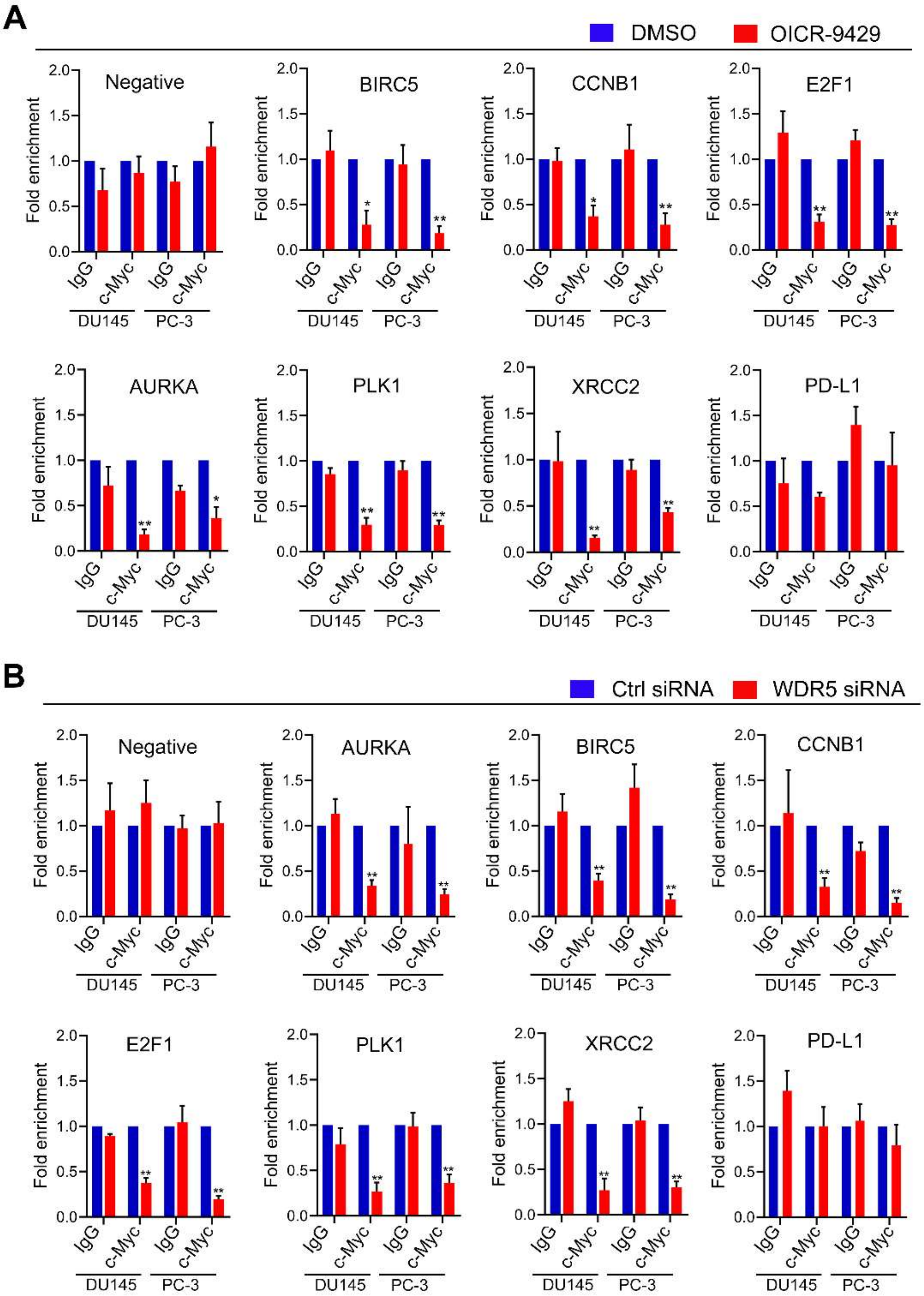

Figure S14. Both OICR-9429 and WDR5 knockdown reduced the recruitment of c-Myc on the promoter of WDR5 target genes. A. ChIP analysis of IgG, c-Myc status of candidate WDR5 target genes in Du145 and PC-3 cells, treated with OICR-9429 or DMSO. **B**. ChIP analysis of IgG, c-Myc status of candidate WDR5 target genes in DU145 and PC-3 cells, treated with WDR5 siRNA or control. The values are normalized to input and presented as the means ± SD. *$p < 0.05$; **$p < 0.01$.

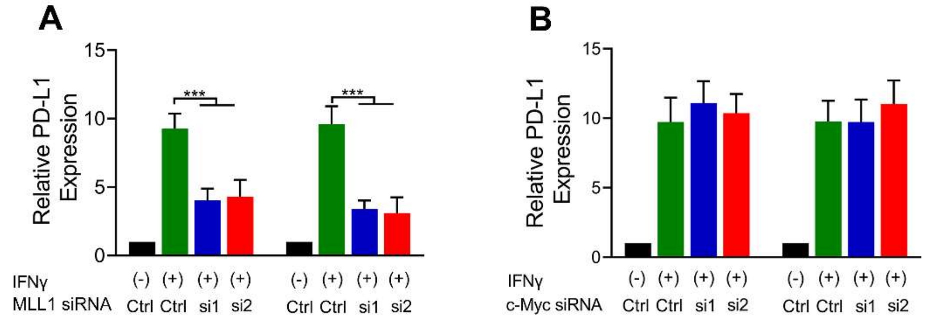

Figure 15. Knockdown of MLL1 (A), but not c-Myc (B) decreased the mRNA level of IFNγ-induced PD-L1 in DU145 and PC-3 cells. The values are normalized to input and presented as the means ± SD. **p* < 0.05; ***p* < 0.01.

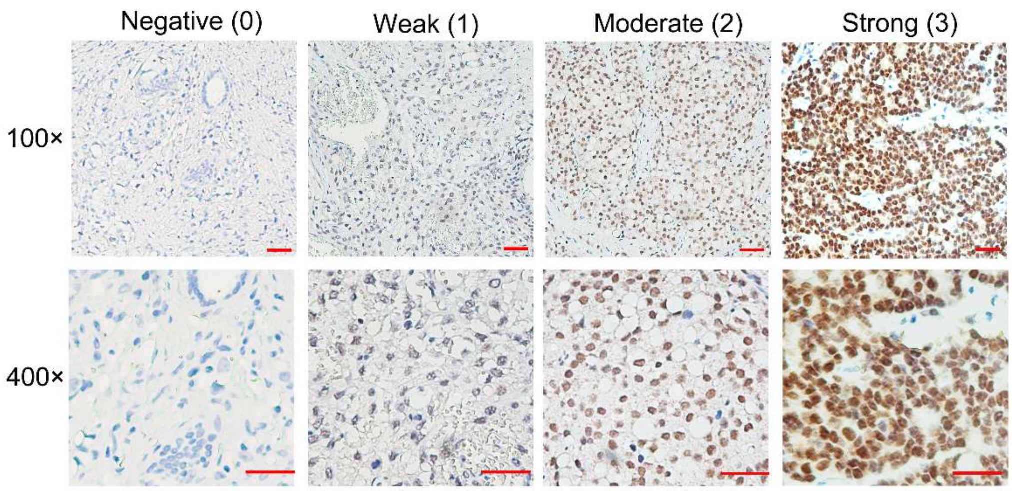

Figure S16. The representative images of different WDR5 expression in PCa by IHC. The level of WDR5 expression was graded as intensity of IHC staining: 0, absent staining; 1, weak staining; 2, moderate staining; 3, strong staining.

Tables

Table S1. Univariate and multivariate analyses of PFS in patients of Cohort 1 and TCGA

Variables	Cohort 1				TCGA			
	Univariate		Multivariate		Univariate		Multivariate	
	HR (95%CI)	*p*-value	HR (95%CI)	*p*-value	HR (95%CI)	*p*-value	HR (95%CI)	*p*-value
Age (y) ≥60/<60	0.87 0.43-1.75	0.690	-	-	0.88 0.46-1.65	0.681	-	-
Gleason score 7(4+3)-10/6-7(3+4)	2.34 1.22-4.49	0.010	1.19 0.55-2.56	0.659	2.49 1.18-5.27	0.017	1.48 0.65-3.38	0.349
Tumor stage T3-4/T2	2.02 1.07-3.81	0.030	1.12 0.57-2.21	0.744	2.47 1.16-5.22	0.019	1.89 0.83-4.33	0.130
Nodal metastasis N1/N0	4.28 2.20-8.33	<0.001	3.37 1.52-7.46	0.003	2.74 1.35-5.57	0.005	1.77 0.83-3.77	0.138
WDR5 high/low	3.26 1.59-6.69	0.010	2.81 1.34-5.91	0.006	3.00 1.51-5.95	0.002	2.73 1.36-5.49	0.005

Table S2. Univariate and multivariate analyses of OS in patients of Cohort 1 and Cohort 2

Variables	Cohort 1				Cohort 2			
	Univariate		Multivariate		Univariate		Multivariate	
	HR (95%CI)	*p*-value	HR (95%CI)	*p*-value	HR (95%CI)	*p*-value	HR (95%CI)	*p*-value
Age (y) ≥60/<60	1.39 0.62-3.10	0.420	-	-	2.50 0.34-18.54	0.369	-	-
Gleason score 7(4+3)-10/6-7(3+4)	2.50 1.25-5.01	0.010	1.29 0.58-2.84	0.535	8.35 2.49-28.09	0.001	4.77 1.33-14.05	0.016
Tumor stage T3-4/T2	2.09 1.06-4.09	0.032	1.26 0.61-2.61	0.541	6.39 2.73-14.97	<0.001	4.49 1.86-10.85	0.001
Nodal metastasis N1/N0	4.28 2.11-8.66	<0.001	3.28 1.45-7.40	0.004	8.42 2.40-29.53	0.001	7.22 1.71-30.39	0.007
WDR5 high/low	3.00 1.41-6.40	0.004	2.41 1.09-5.29	0.029	3.41 1.52-7.64	0.003	1.78 0.69-4.55	0.231

Table S3. The clinicopathological characteristics of patients in present study

Variables		Cohort 1	Cohort 2	TCGA
		Number of cases (%)	Number of cases (%)	Number of cases (%)
Age(y)				
	<60	32 (23.5%)	12 (9.5%)	113 (44.3%)
	≥60	104 (76.5%)	114 (90.5%)	142 (55.7%)
Gleason score	6-7(3+4)	73 (53.7%)	62 (49.2%)	110 (43.1%)
	7(4+3)-10	63 (46.3%)	64 (50.8%)	145 (56.9%)
T stage	T2	78 (57.4%)	90 (71.4%)	105 (41.2%)
	T3	36 (26.5%)	30 (23.8%)	146 (57.3%)
	T4	22 (16.1%)	6 (4.8%)	4 (1.5%)
N status	N0	118 (86.8%)	122 (96.8%)	222 (87.1%)
	N1	18 (13.2%)	4 (3.2%)	33 (12.9%)
WDR5	Low	67 (49.3%)	88 (69.8%)	141 (55.3%)
	High	69 (50.7%)	38 (30.2%)	114 (44.7%)

Patients without available clinical data, including age, Gleason score, T stage and N status, were excluded for the univariate and multivariate analyses in TCGA.

Table S4. The siRNA and shRNA sequences of WDR5 and negative control are listed as follows.

Primer Name	Sequence 5'-3'
WDR5-si1-sence	GCUCAGAGGAUAACCUUGUTT
WDR5-si1-antisence	ACAAGGUUAUCCUCUGAGCTT
WDR5-si2-sence	CCCAGUCCAACCUUAUUGUTT
WDR5-si2-antisence	ACAAUAAGGUUGGACUGGGTT
MLL1-si1-sence	GCACUGUUAAACAUUCCACUUTT
MLL1-si1-antisence	AAGUGGAAUGUUUAACAGUGCTT
MLL1-si2-sence	CCCAUCCAGAACCAGAAGUAUTT
MLL1-si2-antisence	AUACUUCUGGUUCUGGAUGGGTT
c-Myc-si1-sence	CCCAAGGUAGUUAUCCUUAAATT
c-Myc-si1-antisence	UUUAAGGAUAACUACCUUGGGTT
c-Myc-si2-sence	CCUGAGACAGAUCAGCAACAATT
c-Myc-si2-antisence	UUGUUGCUGAUCUGUCUCAGGTT
Negative control- sence	UUCUCCGAACGUGUCACGUTT
Negative control- antisence	ACGUGACACGUUCGGAGAATT

Table S5. The primers used in real time qPCR are listed as follows.

Primer Name	Sequence 5'-3'
WDR5 Forward	AATTCAGCCCGAATGGAGAGT

WDR5 Reverse	AGGCTACATCGGATATTCCCAG
AURKA Forward	CAAATGCCCTGTCTTACTGTC
AURKA Reverse	ATGGAGCATGTACTGACCACC
BIRC5 Forward	CCACTGAGAACGAGCCAGACTT
BIRC5 Reverse	GTATTACAGGCGTAAGCCACCG
CCNB1 Forward	TAAGGCGAAGATCAACATGG
CCNB1 Reverse	TTACCAATGTCCCCAAGAGC
CDK1 Forward	GGAAACCAGGAAGCCTAGCATC
CDK1 Reverse	GGATGATTCAGTGCCATTTTGCC
E2F1 Forward	GGACCTGGAAACTGACCATCAG
E2F1 Reverse	CAGTGAGGTCTCATAGCGTGAC
PLK1 Forward	CCGCCCAACCATTAACGAGCT
PLK1 Reverse	ACCTTGGTGGAATGGTCAGGC
MCM2 Forward	TGCCAGCATTGCTCCTTCCATC
MCM2 Reverse	AAACTGCGACTTCGCTGTGCCA
TopBP1 Forward	TGTGACCCTTTTAGTGGCGTT
TopBP1 Reverse	CTCTTGGGACACATCGCTGG
XRCC2 Forward	TCTGTTTGCTGATGAAGATTCACC
XRCC2 Reverse	CATCGTGCTGTTAGGTGATAAAGC
MLL Forward	TAGTGAGCCCAAGAAAAAGCA
MLL Reverse	TGGAGAGAGTGCTGAGGATGT
c-Myc Forward	CCACACATCAGCACAACTACG
c-Myc Reverse	AAGCTCCGTTTTAGCTCGTTC
GPADH Forward	CAAGGCTGAGAACGGGAAG
GPADH Reverse	TGAAGACGCCAGTGGACTC

Table S6. The primers used in ChIP-real time qPCR are listed as follows.

Primer Name	Sequence 5'-3'
PLK1-P Forward	GAATTCCTCCTCTCTCGGGG
PLK1-P Reverse	TTTAAAATCCAAACCCGCCCG
AURKA-P Forward	CCTGATTGGGTTTCTAGTCCTCC
AURKA-P Reverse	GCCCTTAACAGCTCTGAGACA
E2F1-P Forward	CTGAGGCCTGGGTGATTTATTT
E2F1-P Reverse	CCTCTCCCATCTCATATCCATCC
CCNB1-P Forward	CTTGCCCGGCTAACCTTTC
CCNB1-P Reverse	GAATGCGTTTCCAGGGCGAT
BIRC5-P Forward	CACCACGCCCAGCTAATTTT

BIRC5-P Reverse	AGCATCACTTGAGTCCTGGAG
CDK1-P Forward	AGAAGAACGGAGCGAACAGTA
CDK1-P Reverse	TAGAGCGCGAAAGAAAGAGGA
XRCC2-P Forward	GGCTAACTGTTGAGAACGATGT
XRCC2-P Reverse	TACCCCGAGATAACTTTGCCA
CD274-P Forward	AAACTGGATTTGCTGCCTTGG
CD274-P Reverse	GGAACAACGCTCCCTACCT
Negative control F	GTAATCAGGAAACTGCATAC
Negative control R	CTCAAGACTCAATAGTGATC
